# Peroxisome Proliferator-Activated Receptors and the Hallmarks of Cancer

**DOI:** 10.3390/cells11152432

**Published:** 2022-08-05

**Authors:** Nicole Wagner, Kay-Dietrich Wagner

**Affiliations:** CNRS, INSERM, iBV, Université Côte d’Azur, 06107 Nice, France

**Keywords:** PPAR, cell proliferation, angiogenesis, cellular metabolism, immune surveillance, metastasis, resistance to cell death, tumor growth suppressors

## Abstract

Peroxisome proliferator-activated receptors (PPARs) function as nuclear transcription factors upon the binding of physiological or pharmacological ligands and heterodimerization with retinoic X receptors. Physiological ligands include fatty acids and fatty-acid-derived compounds with low specificity for the different PPAR subtypes (alpha, beta/delta, and gamma). For each of the PPAR subtypes, specific pharmacological agonists and antagonists, as well as pan-agonists, are available. In agreement with their natural ligands, PPARs are mainly focused on as targets for the treatment of metabolic syndrome and its associated complications. Nevertheless, many publications are available that implicate PPARs in malignancies. In several instances, they are controversial for very similar models. Thus, to better predict the potential use of PPAR modulators for personalized medicine in therapies against malignancies, it seems necessary and timely to review the three PPARs in relation to the didactic concept of cancer hallmark capabilities. We previously described the functions of PPAR beta/delta with respect to the cancer hallmarks and reviewed the implications of all PPARs in angiogenesis. Thus, the current review updates our knowledge on PPAR beta and the hallmarks of cancer and extends the concept to PPAR alpha and PPAR gamma.

## 1. Introduction

In addition to receptors for steroid and thyroid hormones, vitamin D and retinoids, and several orphan receptors, peroxisome proliferator-activated receptors (PPARs) belong to the group of nuclear receptors [1,2]. Although peroxisome proliferation in response to hypolipidemic fibrate drugs (PPAR alpha agonist) was described already in 1970s [3,4], it took nearly 20 years for PPAR alpha (PPARα), PPAR beta/delta (PPARβ/δ), and PPAR gamma (PPARγ) to be identified [5,6,7]. On the molecular level, PPARs activate/repress target genes as heterodimers with retinoic X receptors (RxR), which exist in three different isoforms. Liver X receptor α (LxRα) and retinoic acid receptors (RAR)s also form heterodimers with RxR. Thus, depending on the level of expression of the different receptors, the outcome of PPAR activation might differ between cell types (reviewed in [1]). In addition to the classical PPAR/RxR transcriptional complexes [8], PPARs might also interact with glucocorticoid receptors, photoreceptor-specific nuclear receptors, and estrogen-related receptors, which could additionally modify the responses of PPAR activation [9]. As a general PPAR response element, a direct repeat of the sequence AGGTCA, spaced by a single nucleotide, has been originally identified (DR1); in fact for PPAR alpha only [10]. Binding exclusively to this element would not explain the specificity of the identified PPAR alpha, beta/delta, and gamma target genes. Furthermore, thousands of these elements are found in the genome, mostly far away from the gene promoter regions. Experimental evidence suggests a higher heterogeneity of binding elements for PPARs [1,11]. The ligand-dependent and ligand-independent effects, posttranscriptional modifications, co-activators, and co-repressors of PPARs have been extensively reviewed [1,12,13].

Endogenous ligands for PPARs include unsaturated fatty acids, eicosanoids, prostaglandins, and prostacyclins [1,14]. Synthetic activators and inhibitors for all PPARs are available. Until now, only PPARα agonists (e.g., fibrates) have been in clinical use for lipid lowering, the prevention of atherosclerosis, and cardiovascular disease [15,16], while PPARγ agonists (e.g., thiazolidinediones) lower glucose by increasing insulin sensitivity, mainly in skeletal muscle and adipose tissue [17]. In addition to these “classical” applications for the treatment of metabolism-related diseases and metabolic syndrome, PPARs might be involved in a variety of diseases [18] and PPAR modulators might become interesting candidates for neurodegenerative disorders [19], addiction [20], psychiatric disorders [21,22], hepatic and kidney diseases [12,23,24,25], and autoimmune and inflammatory diseases [16,26,27,28,29]. Importantly, PPARs are also critically involved in cancer. The expression of PPARs has been detected in various cancer types and cancer cell lines, but PPARs also play important roles in the tumor stroma, i.e., cancer-associated fibroblasts, mesenchymal cells, endothelial cells, and macrophages (reviewed in [30]). In addition to cancer cell growth, angiogenesis, and the antitumor immune response play an important role in cancer progression and metastasis [31]. Here, we will use the didactic concept of the “Hallmarks of Cancer” by Hanahan and Weinberg [32,33,34,35,36,37] to delineate the functions of the different PPARs in cancer hallmark capabilities. We already used this concept for PPARβ/δ [18,38]. Thus, here, we will describe PPARα and PPARγ functions with respect to the hallmarks of cancer and updates for PPARβ/δ.

## 2. PPARs and Cell Proliferation

### 2.1. PPARα

PPARα expression has been demonstrated in human breast cancer cell lines, which showed increased proliferation upon PPARα activation [39] (Table 1). Leptin and glucose treatment stimulated breast cancer proliferation, which was accompanied by an upregulation of PPARα, suggesting the involvement of PPARα in this process [40]. Similarly, arachidonic acid (AA) has been found to promote breast cancer cell proliferation through the activation of PPARα [41]. However, contrasting results were obtained by another group [42]. The PPAR agonist fenofibrate reduced the proliferation of triple-negative breast cancer cells [43]. Similar results were obtained with clofibrate in inflammatory breast cancer cell lines [44]. Different outcomes on breast cancer cell proliferation may be explained by the different types of breast cancer cell lines used, but also by the different concentrations of fibrates. Tauber and colleagues reported stimulation of the proliferation of MCF-7 breast cancer cells with low fibrate concentrations, and suppression with high doses [45]. Dose-dependent effects of fibrates on cell proliferation have also been reported for human liver cancer cells [46]. The sustained activation of PPARα leads to liver tumorigenesis in rodents. However, in a PPARα humanized model, sustained PPARα activation very rarely provoked liver cancers, which suggests that structural differences between human and mouse PPARα are responsible for the differential susceptibility to peroxisome proliferator-induced hepatocarcinogenesis [47]. In an excellent study, Tanaka and colleagues provided evidence that the hepatitis C virus (HCV) core protein induces heterogeneous activation of PPARα in transgenic mice. The stabilization of PPARα through interaction with the Hepatitis C virus (HCV) core protein and an increase in non-esterified fatty acids, serving as endogenous PPARα ligands, were suggested to contribute to the age-dependent and multicentric hepatocarcinogenesis mediated by the core protein [48]. Interestingly, the hepatocyte restricted the constitutive activation of the PPARα-induced proliferation of hepatocytes, but not carcinogenesis, indicating that the PPARα activation of other cell types than hepatocytes is responsible for the carcinogenic effect of PPARα activation [49]. The existence of an alternatively spliced transcript variant (PPARA-tr) in humans, but not in rodents, with a deficient ligand-binding domain that is unable to bind to peroxisome proliferator-responsive DNA elements (PPREs) could partially explain the species differences in hepatocarcinogenesis [50,51]. A later study suggested a higher susceptibility of PPARα-knockout mice to diethylnitrosamine (DEN)-induced hepatocellular carcinoma (HCC) [52]. However, Kaipainen and colleagues evidenced a tumor-suppressive phenotype in PPARα-deficient mice. The absence of PPARα switches tumor-associated inflammation into tumor-suppressive inflammatory infiltrates, which inhibit tumor angiogenesis and tumor progression independently of the cellular tumor type [53]. Later, PPARα deficiency was also proposed to impair regulatory T-cell functions, leading to the inhibition of melanoma growth [54]. These studies confirm the importance of the molecular properties of stromal host cells for cancer progression, which also explains the differential outcomes of analyses in pure in vitro studies, leading to potential false therapeutic deductions. The PPARα agonist fenofibrate, for example, decreased endometrial cancer cell proliferation in vitro but failed to improve outcomes in vivo [55]. Yokoyama and co-workers reported an inhibition of proliferation in ovarian cancer cell lines in vitro, as well as a reduction in ovarian cancer cell tumor growth in vivo via the activation of PPARα with clofibrate [56]. PPARα is expressed in medulloblastoma cells, and PPARα activation with fenofibrate inhibited cell proliferation in medulloblastoma cell lines [57]. Similar results were proposed using fenofibrate treatment in a glioblastoma cell line [58] and neuroblastoma cells [59]. However, the overexpression of PPARα in glioma stem cells (GSCs) has been observed. GSCs are responsible for tumor initiation, treatment resistance, and recurrence. The knockdown (KD) of PPARα reduced the proliferative and tumor-forming capacities of GSCs, and xenografts failed to establish viable intracranial tumors [60]. PPARα was found to induce carnitine palmitoyltransferase 1C (CPT1C) in a breast and a pancreatic cancer cell line, leading to the activation of cell proliferation [61]. Using syngenic implantation of B16 melanoma, LLC1 lung carcinoma, and SKOV-3 ovarian cancer xenograft models, the efficiency of the tumor growth-inhibiting properties of the PPARα antagonist NXT629 has been demonstrated [62]. Li and colleagues showed that the level of PPARα and its activity were increased in 4-(methylnitrosamino)-l-(3-pyridyl)-lbutanone (NNK)-induced mouse-lung tumors. An increase in PPARα occurred before the formation of lung tumors, indicating that the molecular changes play a role in lung carcinogenesis [63]. In contrast, in two lung cancer cell lines, fenofibrate reduced cell proliferation [64]. PPARα activation in vivo using Wy-14,643 or bezafibrate reduced non-small-cell lung cancer (NSCLC) growth through the inhibition of a proangiogenic epoxygenase. Epoxygenases oxidize arachidonic acid to epoxyeicosatrienoic acids (EET), pro-angiogenic lipids which support tumor growth [65]. Although PPARα activation by Wy-14,643 did not alter proliferation of cancer cell lines in vitro, it reduced tumorigenesis in vivo through the inhibition of angiogenesis [66]. The PPARα agonist fenofibrate has further been demonstrated to suppress B cell lymphoma in mice through the modulation of lipid metabolism. B cell tumors trigger systemic lipid mobilization from white adipose tissue to the liver and increase very-low-density lipoprotein (VLDL)/low-density lipoprotein (LDL) release from the liver to promote tumor growth. B cell lymphoma cells express extremely low levels of PPARα; therefore, fenofibrate did not increase lipid utilization in the tumors but enhanced the clearance of lipids and blocked hepatic lipid release, leading to reduced tumor growth [67]. Fenofibrate has also been proposed to suppress colon cancer cell proliferation in vitro and in in vivo xenograft models through epigenetic modifications involving the inhibition of DNA Methyltransferase 1 (DNMT1) [68]. To summarize, given the highly controversial results regarding the tumor-suppressing or -promoting effects of therapeutic PPARα modulation, especially activation, this intervention seems to be inadequate in the context of cancer. To the best of our knowledge, no clinical trials for the use of PPARα agonists in cancer therapy exist. One trial with the PPARα antagonist TPST-1120 as a monotherapy, and in combination with Nivolumab, Docetaxel or Cetuximab, in subjects with advanced cancers (NCT03829436) is ongoing.

### 2.2. PPARβ/δ

PPARβ/δ expression has been reported in a variety of cancer tissues and cell lines. The effects of PPARβ/δ on cell proliferation and tumor growth are highly controversial, and have been reviewed recently; summarizing tables are provided [38]. Many studies focused on colon cancer. The discrepancy between the observed effects of PPARβ/δ activation can only lead to the conclusion that any therapeutical use of PPARβ/δ modulation has to be avoided. Most studies report a colon cancer-enhancing effect of PPARβ/δ. Examination of PPARβ/δ in human multistage carcinogenesis of the colorectum revealed that its expression increased from normal mucosa to adenomatous polyps to colorectal cancer. The most elevated PPARβ/δ levels were observed in colon cancer cells with a highly malignant morphology [70]. PPARβ/δ expression in human colon cancer tissues was associated with poor prognosis and a higher metastatic risk [71]. An opposite report has been published for human and mouse colon cancer samples; however, no histomorphological detection analysis of PPARβ/δ has been performed to allow for the correlation of PPARβ/δ with expression in malignant cancer cells [72]. It has been demonstrated that PPARβ/δ mediates mitogenic vascular endothelial growth factor (VEGF) release in colon cancer [73,74,75], although one report also claimed that a loss of PPARβ/δ would enhance vascular endothelial growth factor (VEGF) release [76]. PPARβ/δ has been shown to promote [73,77,78,79,80,81,82] or to inhibit [76,83,84] colon cancer in vivo. In line with a pro-tumorigenic role, PPARβ/δ activation via a high-fat diet (HFD) or PPARβ/δ agonist treatment allowed stem and progenitor cells to initiate tumorigenesis in the setting of a loss of the adenomatous polyposis coli (APC) tumor-suppressor gene [85]. PPARβ/δ-mediated epithelial hyperproliferation, which increases the risk for gastric adenocarcinoma, was further found to be induced by Helicobacter pylori infection [86]. Regarding breast cancer, most studies suggest a pro-tumorigenic function of PPARβ/δ. Only two in vitro studies from the same group using the same breast cancer cell line suggest a reduction in cell proliferation upon PPARβ/δ activation [87,88]. The same group published two very similar studies, one using neuroblastoma cell lines, and the other testicular embryonal carcinoma cells, in which PPARβ/δ overexpression and/or activation had beneficial tumor-cell proliferation- or growth-inhibiting effects [89,90]. In contrast, by applying a variety of different molecular tools as either overexpression or knockout models, or conducting pharmacological activation or inhibition of PPARβ/δ, it has been shown, in vivo, that PPARβ/δ favors mammary tumorigenesis [91,92,93,94]. 3-phosphoinositide-dependent kinase-1 (DK1) favors these tumorigenic properties of PPARβ/δ in breast cancer [92,93]. Fatty-acid-binding protein 5 (FABP5), which shuttles ligands from the cytosol to PPARβ/δ, underlines the importance of endogenous PPARβ/δ ligands for cancer growth, as knockout of FABP5 was sufficient to reduce mammary tumorigenesis [95]. In line with this, FABP5 has been shown to convert the strong anticarcinogenic properties of retinoic acid (RA) into tumor-promoting functions as it delivers RA to the mitogenic and anti-apoptotic PPARβ/δ receptor [96]. Similar to the effects observed in mammary carcinomas, activation of the FABP5/PPARβ/δ pathway was shown to promote cell survival, proliferation, and anchorage-independent growth in prostate cancer cells [97]. The oncogenic redirection of transforming growth factor (TGF)-β1 signaling via the activation of PPARβ/δ was also identified to promote prostate cancer growth [98]. One study, however, suggested the inhibition of prostate cancer growth by PPARβ/δ through a noncanonical and ligand-independent pathway [99]. The activation of PPARβ/δ has been proposed to inhibit liver tumorigenesis in hepatitis B transgenic mice [100]; however, in different human hepatocellular carcinoma cell lines, the activation of PPARβ/δ enhanced the growth of these cancer cells through the activation of cyclooxygenase (COX)-2 [101]. PPARβ/δ activation has been shown to inhibit melanoma skin cancer cell proliferation through repression of the Wilms tumor suppressor (WT)1 [102], which favors human melanoma progression [103]. PPARβ/δ-knockout animals were more susceptible to skin carcinogenesis as their wildtype counterparts and PPARβ/δ agonists inhibited keratinocyte proliferation [104], as well as proliferation in a human squamous-cell carcinoma cell line [105]. In line with these finding, the authors proposed a protective effect of PPARβ/δ activation, coupled with the inhibition of COX-2 activity, to increase the efficacy of chemoprevention in skin tumorigenesis [106,107]. However, a later report from this group showed that PPARβ/ δ is not involved in the suppression of skin carcinogenesis by non-steroidal anti-inflammatory drugs (NSAID) which inhibit COX-2 [108]. In contrast to an inhibitory function of PPARβ/ δ in the tumorigenesis of non-melanoma skin cancers, one study clearly evidenced the pro-tumorigenic role of PPARβ/δ involving the direct activation of proto-oncogene tyrosine-protein kinase Src, which promotes the development of ultraviolet (UV)-induced skin cancer in mice [109]. An elegant study focused on the importance of fibroblast PPARβ/ δ expression in non-melanoma skin tumorigenesis. Although the chemically induced skin tumors of animals with the conditional deletion of PPARβ/ δ in fibroblasts showed increased proliferation, the tumor burden was smaller and the tumor onset delayed; this indicates the role of fibroblast PPARβ/δ in epithelial–mesenchymal communication, which further influences tumor growth [110]. Regarding lung cancer, high expression of PPARβ/δ limited to cancer cells has been demonstrated in human cancer samples. In lung cancer cell lines, the activation of PPARβ/δ stimulated proliferation and inhibited apoptosis [111,112]. Nicotine increases PPARβ/δ expression in lung carcinoma cells, which contributes to increased proliferation [113]. In contrast, one study using the activation of PPARβ/δ in two lung cancer cell lines in vitro did not find differences for proliferation upon stimulation of PPARβ/δ [114]. In transgenic mice lacking one or both PPARβ/δ alleles, the growth of RAF-induced lung adenomas was decreased [115]. Although cell proliferation in mouse LLC1 lung cancer cells was decreased upon activation of PPARβ/δ, LLC1 tumor growth in vivo was enhanced in mice with conditional vascular overexpression of PPARβ/δ, underlining the importance of crosstalk between the tumor stroma and cancer cells for tumor growth [11]. One study reported that PPARβ/δ activation promoted apoptosis and reduced the tumor growth of nasopharyngeal carcinoma cells [116]. PPARβ/δ was found to be highly expressed in liposarcoma compared to benign lipoma, and PPARβ/δ activation increased liposarcoma cell proliferation, which was mediated via the direct transcriptional repression of leptin by PPARβ/δ [117]. Additionally, in thyroid tumors, PPARβ/δ was increased and correlated with the expression of the proliferation marker Ki67. PPARβ/δ activation increased the cell proliferation of thyroid cells [118]. PPARβ/δ was highly expressed in epithelial ovarian cancer cell lines and the inhibition of PPARβ/δ reduced their proliferation and tumor growth in vivo. Interestingly, aspirin, a NSAID that preferentially inhibits COX-1, compromised PPARβ/δ function and cell growth by inhibiting extracellular signal-regulated kinases 1/2 [119]. PPARβ/δ promoted the survival and proliferation of chronic lymphocytic leukemia cells [120] and changed the outcome of signaling from cytokines such as interferons (IFNs) [121]. A detailed table on the effects of PPARβ/δ on cell proliferation and tumor growth can be found in [38]. In conclusion, most studies identified PPARβ/δ as a tumor-promoting factor which increases cell proliferation and cancer growth. Although some studies report the inhibition of cancer cell proliferation upon PPARβ/δ activation, the therapeutic modulation of PPARβ/δ appears dangerous. Consequently, no cancer-related clinical trials are reported.

### 2.3. PPARγ

PPARγ expression is found in a variety of cancer tissues and cell lines. The activation of PPARγ by different agonists increased the frequency and size of colon tumors in C57BL/6J-APCMin/+ mice [122,123] (Table 2). However, in human colon cancer cell lines, PPARγ inhibited tumor-cell proliferation [124,125,126,127]. Prostate cancers were found to overexpress PPARγ. The PPARγ agonist troglitazone inhibited the proliferation of PC-3 prostate cancer cells in vitro and in xenograft models in vivo [128], which was confirmed by others in later studies [129,130]. Similarly, growth inhibition via PPARγ activation has been described for liposarcoma [131], gastric cancer [132,133], bladder carcinoma [130,134], renal cell carcinoma [130], neuroblastoma [135,136], glioblastoma [137,138], melanoma [139,140,141,142], NSCLC [143,144], adrenocortical cancer [145,146], hepatocellular carcinoma [147], endometrial carcinoma [148], ovarian cancer [149,150], multiple myeloma [151], B cell lymphoma [152], mesothelioma [153], and esophageal squamous-cell carcinoma [154]. Most of these studies used cancer cell lines and PPARγ agonist treatment in vitro. Exciting results for therapeutic effects of PPARγ activation have been obtained in chronic myeloid leukemia (CML). With standard therapies, mainly tyrosine kinase inhibitors (TKIs), only 10% of patients achieve a complete molecular response/remission (CMR). This is mainly due to a pool of quiescent CML leukemia stem cells (LSCs), which are not completely eradicated by TKIs. Prost and colleagues demonstrated that thiazolidinediones target this pool of LSCs through the decreased transcription of signal transducer and activator of transcription (STAT) 5, leading to sustained CMR in a small group of patients [155]. A proof-of-concept study including 24 patients yielded positive outcomes with a combined therapy of pioglitazone and imatinib (TKI) [156]. A phase 2 trial is ongoing (EudraCT 2009-011675-79). PPARγ has been identified as a critical modifier in thyroid carcinogenesis using transgenic animals harboring a knock-in dominant-negative mutant thyroid hormone receptor beta (TRbetaPV/PV mouse), which spontaneously develop follicular thyroid carcinoma. TRbetaPV/PV mice were crossed with PPARγ +/− mice, and it was shown that thyroid carcinogenesis progressed faster in animals with PPARγ haplo-insufficiency. Reduced PPARγ led to the activation of the nuclear factor-kappaB signaling pathway, resulting in the repression of apoptosis. Furthermore, the treatment of TRbetaPV/PV mice with rosiglitazone delayed the progression of thyroid carcinogenesis by decreasing cell proliferation [157]. Wu and colleagues showed that the inhibition of PPARγ via the overexpression of dominant negative PPARγ (dnPPARγ) in the myeloid cell lineage provokes systemic inflammation and an increase in myeloid-derived suppressor cells (MDSC), which led to immunosuppression and the appearance of multiple cancers [158]. In breast cancer [159,160] and uterine leiomyomas [161], the growth-inhibiting effect of PPARγ activation was attributed to the inhibition of estrogen-receptor signaling. This seems to be partially mediated through the repression of leptin’s stimulatory effects on estrogen signaling by PPARγ [162]. However, later, it was shown that the PPARγ agonist prostaglandin 15-deoxy-Δ^12,14^-PGJ2 (15d-PGJ2) inhibits the transcriptional activity of estrogen receptor alpha via PPARγ-independent covalent modification of its DNA-binding domain [163]. Methylene-substituted diindolylmethanes (C-DIMs) are PPARγ-activating agents. They reduce the proliferation of breast cancer cell lines. However, the decrease in cell growth was not inhibited by PPARγ antagonists, indicating that the observed effect might be PPARγ-independent [164]. An elegant study used transgenic mice prone to mammary-gland cancer crossed with mice expressing a constitutively active form of PPARγ in the mammary gland. The resulting PyV/VpPPARγ females developed tumors with accelerated kinetics. Even before reaching maturity at around 30 days of age, female mice displayed palpable tumor masses. These results indicate that once an initiating event has taken place, increased PPARγ signaling exacerbates mammary-gland tumor development [165]; this is similar to the observed situation of accelerated colon cancer formation in APCMin/+ mice treated with thiazolidinediones described before [122,123]. Avena and colleagues focused on the importance of the tumor stroma for cancer growth. They demonstrated that the overexpression of PPARγ in breast cancer cells reduced tumor growth in a xenograft model and demonstrated increased autophagy in the tumor cells. However, when breast cancer cells were co-injected with PPARγ-overexpressing fibroblasts, tumor growth was significantly increased. Stromal cells with overexpression of PPARγ displayed metabolic features of cancer-associated fibroblasts, with increased autophagy, glycolysis, and senescence; this supports a catabolic pro-inflammatory microenvironment that metabolically enhances cancer growth. The activation of an autophagic program, therefore, have pro- or antitumorigenic effects, depending on the cellular context [166]. The mammary secretory-epithelial-cell-specific knockout of PPARγ enhanced tumor growth in a 7,12-dimethylbenz[a]anthracene (DMBA)-induced breast cancer model [167]. A small clinical trial in patients with early-stage breast cancer did not evidence differences in breast tumor-cell proliferation upon treatment with rosiglitazone, administered between the time of diagnostic biopsy and definitive surgery [168]. PPARγ ligands did not prevent chemically or UV-induced skin tumors, although they significantly inhibited basal-level keratinocyte proliferation [169].

It is important to note that the anti-cancer effects of thiazolidinediones (rosiglitazone, pioglitazone, and troglitazone) might be independent of PPARγ activation, as it has been demonstrated that they are mediated by translation inhibition [170]. In osteosarcoma cell lines, troglitazone enhanced proliferation in one study [171], and inhibited proliferation in another [172]. Srivastava and colleagues demonstrated, in a lung cancer model, that treatment with the PPARγ agonist pioglitazone triggers a metabolic switch that inhibits pyruvate oxidation and reduces glutathione levels. These metabolic changes increase reactive oxygen species (ROS) levels, which leads to the rapid hypophosphorylation of the retinoblastoma protein (RB) and cell-cycle arrest [173]. In a very recent study, Musicant and colleagues demonstrated that the inhibition of PPARγ might be beneficial in mucoepidermoid carcinoma (MEC), a salivary-gland cancer that is driven primarily by a transcriptional coactivator fusion composed of cyclic AMP-regulated transcriptional coactivator 1 (CRTC1) and mastermind-like 2 (MAML2). The chimeric CRTC1/MAML2 (C1/M2) oncoprotein induces transcriptional activation of the non-canonical peroxisome proliferator-activated receptor gamma coactivator-1 alpha (PGC-1α) splice variant PGC-1α4, which regulates PPARγ-mediated insulin-like growth factor (IGF) 1 expression. The inhibition of PPARγ by inverse agonists inhibits MEC cell proliferation and tumor growth in xenograft models [174]. Besides the clinical trials already mentioned, one trial (NCT00408434) of efatutazone in patients with advanced solid malignancies and no curative therapeutic options reported evidence of disease control [175]. In other clinical trials investigating the effects of efatutazone in combination with carboplatin/paclitazel in NSCLC (NCT01199055), or in combination with erlotinib (NCT01199068), partial responses were around 40%. However, in a clinical trial for liposarcoma (NCT02249949), efatutazone resulted in neither complete nor partial responses. The development of efatutazone has been discontinued. Clinical trials for pioglitazone in the treatment of leukoplakia in head and neck cancer (NCT00099021) resulted in partial responses of 70%, and in another trial for oral leukoplakia (NCT00951379), partial responses of 46% were achieved. Over twenty years ago, a very small clinical trial in three patients with liposarcoma treated with troglitazone already provided some evidence for adipocytic differentiation and decreased proliferation [176]. However, no results are available for later trials with a higher number of patients enrolled (NCT00003058 and NCT00004180). A table with detailed information regarding clinical trials using PPARγ agonists for cancer treatment is given in [177]. Although a large body of evidence suggests that PPARγ functions as a tumor suppressor, the role of PPARγ in tumorigenesis remains controversial. The predominant use of in vitro cell culture studies is limited in its elucidation of the biological relevance of PPARγ in cancer, as complex gene–gene and gene–environment interactions are not considered. It can be concluded that the role of PPARγ in cancer depends on the specific cancer type, the tumor stage, and the tumor environment, which implies that the therapeutical modulation of PPARγ must be considered with caution.

**Figure 1:**
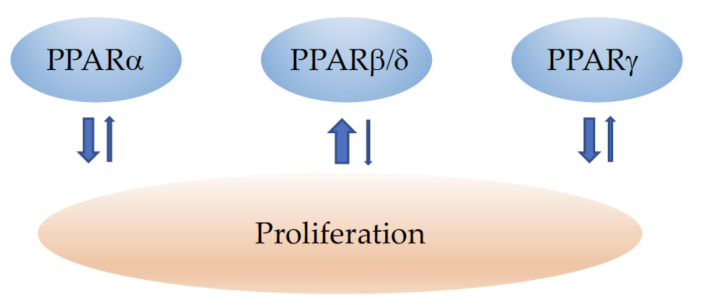
Schematic illustration of the influence of PPARα, PPARβ/δ, and PPARγ on cancer-cell proliferation. ↓ indicates inhibition and ↑ an increase in cell growth and proliferation. The width of the arrows corresponds to the number of studies reporting similar effects. Note that for a certain cancer type, the situation might be different (see the main text for details).

The major effects of PPARα, PPARβ/δ, and PPARγ on proliferation are depicted in Figure 1.

## 3. PPARs and Cell Death

### 3.1. PPARα

The PPARα activator fenofibrate has been shown to induce apoptosis in a human hepatocellular carcinoma cell line through an increase in reactive oxygen species (ROS) [178]. As another molecular mechanism of PPARα-dependent apoptosis, it has been proposed that PPARα serves as an E3 ubiquitin ligase to induce Bcl2 ubiquitination and degradation, leading to apoptosis [179]. Additionally in endometrial cancer [180], breast cancer [181], glioblastoma [182], colon cancer [68,183], ovarian cancer [56], medulloblastoma [57], neuroblastoma [59], pancreatic cancer [184], and NSCLC [185], the activation of PPARα induced apoptosis. These studies were mainly performed using a cancer cell line in in vitro assays. Conjugated linoleic acids induced apoptosis in a variety of human cancer cell lines, which was accompanied by a strong increase in PPARα [186]. The synergistic pro-apoptotic anticancer activity of clioquinol (5-chloro-7-iodo-8-hydroxyquinoline) and docosahexaenoic acid (DHA) in human cancer cells has also been suggested to be mediated by PPARα signaling [187]. Zang and colleagues reported that the dual PPARα/γ agonist TZD18 provoked apoptosis in human leukemia, glioblastoma, and breast cancer cell lines through the induction of the endoplasmic reticulum stress response [188]. Later, the same observations were made in gastric cancer cell lines [189]. However, it is not clear if these actions were mediated through combined PPARα/γ signaling or solely through PPARα or PPARγ signaling. Crowe and colleagues evidenced that combined therapy using PPAR and RXR ligands for breast cancer treatment resulted in growth inhibition. This was due to apoptosis when PPARα ligands were used. In contrast, PPARγ agonists provoked decreased growth characterized by S-phase inhibition [181]. In mantle-cell lymphoma (MCL), a type of aggressive B cell non-Hodgkin’s lymphoma, which is frequently resistant to conventional chemotherapies, fenofibrate efficiently induced apoptosis through the downregulation of tumor necrosis factor (TNF) α. The addition of recombinant TNFα partially rescued fenofibrate-induced apoptosis, whereas the PPARα antagonist GW6471 did not affect the fenofibrate effects. Therefore, it might be possible that fenofibrate induced apoptosis through other mechanisms than the activation of PPARα [190]. In retinoblastoma cells, apoptosis was induced by fatty acid synthase, which led to the downregulation of PPARα; however, the relationship between these molecular events has not been investigated [191]. Similarly, in hepatic carcinoma cells, apoptosis was induced by the flavonoid quercetin, which downregulated PPARα expression [192]. The cause–effect relationship remains to be elucidated. Fenofibrate was found to induce apoptosis in triple-negative breast cancer cell lines, which involved the activation of the nuclear factor ‘kappa-light-chain-enhancer’ of activated B-cell (NF-κB) pathways, as the effect could be almost totally blocked by an NF-κB-specific inhibitor. The induction of apoptosis by fenofibrate was, however, independent of PPARα expression status, as the PPARα antagonist GW6471 did not change apoptosis induction by fenofibrate [43]. In contrast, the induction of apoptosis in hepatocellular carcinoma cells via the overexpression of PPARα was dependent on NF-κB signaling, as PPARα was found to directly interact with IκBα (nuclear factor kappa-light-polypeptide-gene-enhancer in B-cells inhibitor alpha) [52]. In contrast to most studies suggesting a pro-apoptotic function of PPARα activation, Li and coworkers reported that the PPARα inhibitor MT886 induced apoptosis in hepatocarcinoma cell lines, and the agonist fenofibrate significantly increased proliferation, the expression of cell-cycle-related protein (CyclinD1, CDK2), and cell-proliferation-related proteins (PCNA) [46]. Similarly, Abu Aboud and colleagues demonstrated enhanced apoptosis in renal-cell carcinoma upon PPARα inhibition in vitro [193] and in vivo through a decrease in enhanced fatty-acid oxidation and oxidative phosphorylation, and further cancer-cell-specific glycolysis inhibition [194]. The induction of apoptosis via PPARα inhibition has also been described in head and neck paragangliomas (HNPGLs); in one case, the authors described the inhibition of the PI3K/GSK3β/β-catenin signaling pathway as the underlying molecular mechanism [195]. In conclusion, most of the studies suggest that PPARα activation induces apoptosis in cancer cells. However, given that a substantial number of research works also propose the opposite, and advise the use of PPARα inhibition to provoke apoptosis in tumor cells, no clear recommendation for therapeutic PPARα modulation in cancer treatment can be postulated.

### 3.2. PPARβ/δ

The function of PPARβ/δ in cancer-cell death was reviewed in detail in [38]. Most studies support the cell-death-preventing role of PPARβ/δ in tumor cells. In 1999, it was already demonstrated that PPARβ/δ was overexpressed in colorectal cancers (CRC) with adenomatous polyposis coli (APC)/β-catenin mutations, leading to the prevention of apoptosis in colon cancer cells. NSAIDs could compensate for this defect by suppressing PPARβ/δ and promoting apoptosis [196]. Cyclooxygenase-derived prostaglandin E_2_ (PGE_2_), which is overexpressed in most CRCs, was further found to indirectly transactivate PPARβ/δ to inhibit colon cancer-cell apoptosis [197]. Interestingly, it has been demonstrated that fibroblasts isolated from the mucosa of hereditary non-polyposis colorectal cancer (HNPCC) patients produced 50 times more PGE_2_ than normal fibroblasts. Stromal overproduction of PGE_2_ in HNPCC patients is likely to prevent the apoptosis of neoplastic lesions through the activation of PPARβ/δ, thereby facilitating progression into a malignant state [198]. Studies using HCT116 colon cancer cells confirmed that treatment with the PPARβ/δ agonist GW501516 diminished serum-withdrawal-induced apoptosis, which was not the case in PPARβ/δ-deficient HCT116 cells; this indicates the specificity of the apoptosis-preventing effect for PPARβ/δ [77]. Other mechanisms for the PPARβ/δ-mediated prevention of apoptosis in colon cancer have been suggested, such as the activation of the 14-3-3ε protein [199], or survivin [200] expression by PPARβ/δ. In contrast to these studies, one report suggested a pro-apoptotic function of PPARβ/δ in colon carcinoma. GW0742 agonist treatment induced apoptosis in wildtype, but not in PPARβ/δ-knockout animals with chemically induced colon carcinoma. Apoptosis was quantified via TdT-mediated dUTP-biotin nick-end labeling (TUNEL) staining of colon sections and subsequent cell counting; however, as no images were provided, it is difficult to assume TUNEL-specific positivity for cancer cells [83]. A study from the same group using different human colon cancer cell lines treated with hydrogen peroxide to induce apoptosis, different concentrations of the PPARβ/δ agonist GW0742, and NSAIDs could not find evidence for a decrease in apoptosis upon PPARβ/δ activation [72]. Conjugated linoleic acids (CLAs) were found to reduce proliferation in different human cancer cell lines. In cancer cell lines in which the inhibition of cell proliferation was correlated with apoptosis induction, PPARβ/δ expression became strongly downregulated [186]. PPARβ/δ activation decreased human and mouse melanoma cell proliferation; however, no changes in apoptosis could be observed [102]. The activation of PPARβ/δ has been shown to inhibit cisplatin-induced apoptosis in human lung cancer cell lines [111], and the knockout of PPARβ/δ induced apoptosis in lung cancer cells [112]. In mouse LLC1 lung cancer cells, the modulation of PPARβ/δ activity did not influence apoptosis [11]. The inhibition of PPARβ/δ sensitized neuroblastoma cells to retinoic acid-induced cell death [201]. In contrast, in prostate cancer cell lines, ginsenoside Rh2- [202] and telmisartan- [203] induced apoptosis were hampered by the inhibition of PPARβ/δ. In line with a pro-apoptotic function of PPARβ/δ, enhanced apoptosis in a bladder carcinoma cell line [204] as well as in nasopharyngeal tumor cells [116] and liver cancer cells [205] was reported upon PPARβ/δ activation.

### 3.3. PPARγ

Over twenty years ago, Padilla and colleagues already described that 15d-PGJ2 that binds to PPARγ exerts cytotoxicity in malignant B-cell lymphoma via apoptosis induction. Additionally, thiazolidinedione PPARγ agonists negatively affected B-lineage cells, indicating a specific PPARγ function of counteracting the stimulatory effects of prostaglandin E_2_ (PGE_2_) [206,207]. Later, the inhibition of NFκB was shown to be the major mechanism of 15d-PGJ_2_-induced apoptosis in aggressive B-cell malignancies. These effects were mimicked by the proteasome inhibitor MG-132, but not by troglitazone, suggesting that 15d-PGJ2-induced apoptosis is independent of PPARγ [208]. In multiple myeloma, the overexpression of PPARγ induced apoptosis through the inhibition of Interleukin-6 production [151]. Similarly, in acute myeloid leukemia (AML), the forced expression of PPARγ regulated the induction of apoptosis via caspase-8 activation [209]. The activation of PPARγ by 15d-PGJ2 has also been demonstrated to inhibit tyrosine phosphorylation of epidermal growth factor receptors ErbB-2 and ErbB-3 in a breast cancer cell line, leading to a dramatic increase in apoptosis [159]. A later study, however, showed that while 15d-PGJ2 activates PPRE-mediated transcription, PPARγ is not required for 15d-PGJ_2_-induced apoptosis in breast cancer cells. As other possible mechanisms of apoptosis induction by 15d-PGJ2, the inhibition of NFκB-mediated survival pathways, the inhibition of transcriptional activation of COX-2, and the inhibition of the ubiquitin proteosome were proposed [210]. The PPARγ-independent induction of apoptosis by 15d-PGJ_2_ has also been demonstrated in prostate and bladder carcinoma cells [211]. Additionally, 15d-PGJ_2_ induced apoptosis in pancreatic cancer cells through the downregulation of human telomerase reverse transcriptase (hTERT) [212]. Thiazolidinediones sensitize breast cancer cells to tumor necrosis factor-related apoptosis-inducing ligand (TRAIL) therapy by reducing cyclin D3 levels, but not other D-type cyclins [213]. Later, combined treatment with TRAIL and PPARγ ligands, especially 15d-PGJ2, was proposed to overcome chemoresistance in ovarian cancers for successful apoptosis induction [214]. The simultaneous activation of PPARγ and RXR has been suggested to promote apoptosis, implicating the upregulation of p53 in breast cancer cell lines [215]. NSAIDs, considered in cancer prevention due to their inhibitory effect on cyclooxygenases (COX), have recently been proposed to exert their antineoplastic activity through the activation of PPARγ, which induces proline dehydrogenase/proline oxidase (PRODH/POX)-dependent apoptosis in breast cancer cells [216]. In many other studies PPARγ agonists induced apoptosis in bladder cancer [217], gastric carcinoma [133,218], lung cancer [219], esophageal adenocarcinoma [220], pancreatic cancer [221], hepatocellular carcinoma [222], neuroblastoma [223], melanoma [141,142], glioblastoma [224], leukemia [225], leiomyoma [226], mesothelioma [153], and colon carcinoma [227]. Nevertheless, it is not always clear if apoptosis induction is mediated via PPARγ activation. In colon carcinoma, increased PPARβ/δ expression and/or activation of PPARβ/δ antagonized the ability of PPARγ to induce cell death. The activation of PPARγ was found to decrease survivin expression and increase caspase-3 activity, whereas the activation of PPARβ/δ counteracted these effects [200]. A highly interesting study investigated the role of PPARγ coactivator-1 alpha (PGC-1α) in the induction of apoptosis in human epithelial ovarian cancer cells. The overexpression of PGC-1α in human epithelial ovarian cancer cells induced cell apoptosis through the coordinated regulation of Bcl-2 and Bax expression. The suppression of PPARγ expression via siRNA or PPARγ antagonist treatment inhibited PGC-1α-induced apoptosis, suggesting that PPARγ is required for apoptosis induction by PGC-1α [211]. Alternative promoter and mRNA splicing give rise to several PPARγ mRNA and protein isoforms, reviewed in [228]. Kim and coworkers identified a novel splice variant of human PPARγ 1 (hPPAR γ1) that exhibits dominant-negative activity in human tumor-derived cell lines and investigated the function of a truncated splice variant of hPPARγ 1 (hPPARγ1(tr)) in lung cancer. The overexpression of hPPARγ1(tr) rendered cancer cells more resistant to chemotherapeutic drug- and chemical-induced cell death [229]. PPARγ mediated apoptosis induction by n-3 polyunsaturated fatty acids (n-3 PUFA) in a breast cancer cell line, which might explain the beneficial effects of diets enriched in n-3 PUFA [230]. Like the results described above for breast cancer, in colon cancer, the anti-apoptotic activity of the PPARγ agonist troglitazone was also found to be independent of PPARγ. Instead of apoptosis induction through PPARγ, the activation of early growth response-1 (Egr-1) transcription factor was identified as the underlying molecular mechanism [231]. This has also been described for the apoptotic action of C-DIMs, PPARγ agonists, which decreased colon cancer cell survival through the PPARγ-independent activation of early growth response protein (Egr) 1 [127]. In contrast, Telmisartan, an angiotensin II receptor blocker (ARB), was found to inhibit cancer cell proliferation and induce apoptosis through the activation of PPARγ [232,233,234]. In contrast to these pro-apoptotic actions of PPARγ agonists, the PPARγ agonist troglitazone increased cell proliferation and inhibited staurosporine-induced apoptosis in several osteosarcoma cell lines through Akt activation [171]. Later, studies from the Kilgore lab provided evidence that the unreflected therapeutical use of PPARγ ligands in patients predisposed to or already diagnosed with cancer, especially breast cancer, could be dangerous. They identified Myc-associated zinc finger protein (MAZ) as a transcriptional mediator of PPARγ1 expression. The down-regulation of PPARγ1 expression led to reduced cellular proliferation and the induction of apoptosis in breast cancer cells [235]. Interestingly, it has been demonstrated that PPARγ ligands can have distinct activities. One relates to the ability of ligands to act as canonical agonists of the nuclear receptor on peroxisome proliferator response elements, which leads to adipogenesis. The second relates to the allosteric inhibition of phosphorylation of the Ser273 residue of PPARγ. PPARγ is phosphorylated in response to DNA damage, and the inhibition of phosphorylation by novel noncanonical ligands can sensitize cancer cells to DNA-damaging agents. They might represent a safer approach in cancer therapies as the established canonical agonists, which are used less and less frequently due to reported severe side effects or contradictory therapeutical outcomes [236]. A good study by Schaefer and colleagues using hepatocellular carcinoma cells demonstrated that PPARγ antagonists prevented adhesion to the extracellular matrix followed by caspase-dependent apoptosis (anoikis). They found that PPARγ inhibitor T0070907 was significantly more efficient in causing cancer-cell death than the activators troglitazone and rosiglitazone, which had no effect on cell adhesion and caused cell death at much higher concentrations [237]. Later studies confirmed this mechanism of anoikis induction by PPARγ antagonists in squamous-cell carcinoma [178,238]. Some reports evidenced autophagy induction in cancer cells upon PPARγ activation [239,240,241]. Autophagy can either suppress or promote tumor growth [242], and deducing that the induction of autophagy in cancers via PPARγ modulation might be beneficial is, consequently, erroneous. The difficulty in categorizing PPARγ activation in cancer therapy as beneficial or disadvantageous is also well-illustrated in a study from Baron and colleagues, who investigated the effects of ciglitazone in two different colon cancer cell lines: HT29 and SW480 cells. Ciglitazone induced apoptosis in HT29 cells, but stimulated SW480 cell proliferation. The authors concluded that the differential responses for growth regulation result from cell-specific protein synthesis and differences in protein regulation [243]. Based on the outcomes of all these studies, it is therefore impossible to recommend PPARγ modulation to induce cancer-cell death.

**Figure 2:**
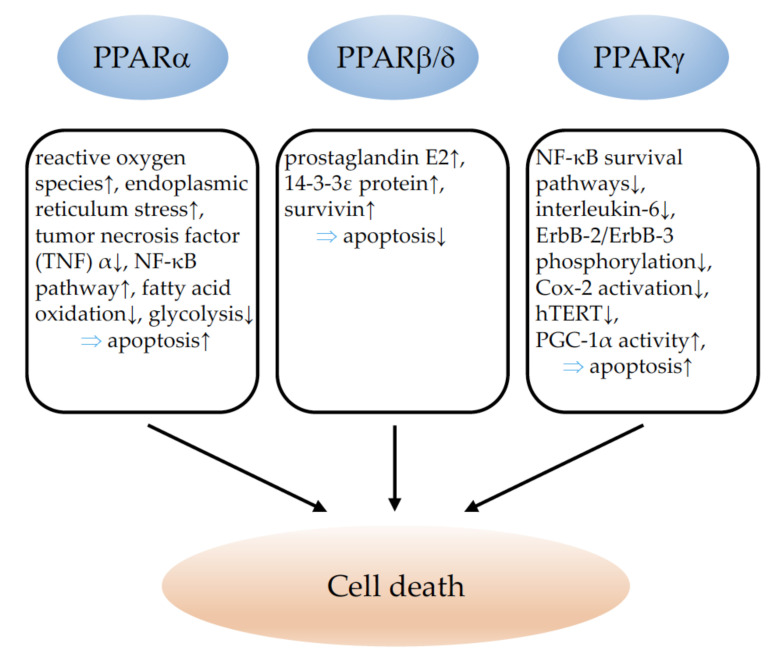
Illustration of the influence of PPARα, PPARβ/δ, and PPARγ on cancer-cell death. ↓ indicates inhibition and ↑ indicates an increase. ⇒: leads to; TNFα: tumor necrosis factor alpha; NF-κB: nuclear factor kappa-light-chain-enhancer of activated B cells; Cox-2: cyclooxygenase-2; hTERT: telomerase reverse transcriptase human; PGC-1α: peroxisome proliferator-activated receptor gamma coactivator-1 alpha.

The major effects of PPARα, PPARβ/δ, and PPARγ on cell death and the underlying molecular mechanisms are summarized in Figure 2.

## 4. PPARs and Angiogenesis

### 4.1. PPARα

The activation of PPARα is, in general, considered to suppress tumor angiogenesis, which has been reviewed in detail in [18,177]. One study investigated the expression of PPARα in human non-melanoma skin cancer and found less expression of PPARα in squamous-cell carcinoma and actinic lesions than in normal human skin samples; however, no correlation with vascular densities could be established [244]. A single study using syngenic tumor inoculation experiments in PPARα knockout mice observed a reduction in tumor vascularization and proposed that PPARα might favor tumor angiogenesis [53]. However, the same research group, as well as the great majority of other laboratories, could establish an anti-angiogenic effect of PPARα activation, implying different PPARα agonists in a variety of animal tumor models [56,65,245,246,247,248].

### 4.2. PPARβ/δ

In contrast to PPARα, PPARβ/δ clearly favors tumor angiogenesis. Studies of human cancer samples revealed that the overexpression of PPARβ/δ in malignant squamous-cell carcinoma [244], pancreatic, prostate, breast cancer, and endometrial adenocarcinoma [249], as well as in colon carcinoma [250], was correlated with higher vessel densities and enhanced tumor progression. Using PPARβ/δ-knockout models, several research groups reported diminished or disturbed tumor-vessel formation and impaired tumor growth upon the induction of different cancer types [249,251,252], confirming the supporting role of PPARβ/δ for tumor angiogenesis and progression. Pharmacological PPARβ/δ activation induced Il-8 and VEGF expression in endothelial cells [253,254]. Enhanced Il-8 expression caused tumor angiogenesis and metastasis formation [252]. Using mice with inducible vascular-specific overexpression of PPARβ/δ [255], it has been demonstrated that the overexpression of PPARβ/δ solely in endothelial cells is sufficient to promote tumor angiogenesis, progression, and metastasis formation. The increased tumor angiogenesis in this model is related to enhanced endothelial Vegf receptor 1, 2, and 3; platelet-derived growth factor receptor beta (Pdgfrβ); platelet-derived growth factor subunit B (Pdgfb); and tyrosinkinase KIT (c-kit) expression [11]. This clearly indicates the danger of the potential therapeutic use of PPARβ/δ agonists, which have been further shown to promote tumor vascularization, growth, and metastasis occurrence [11]. Further detailed information on the angiogenesis-promoting effects of PPARβ/δ can be found in several recent review articles [18,38,177].

### 4.3. PPARγ

PPARγ activation has mostly been considered to inhibit tumor angiogenesis (reviewed in detail in [177]). Although no correlation could be found between PPARγ expression and vascular density in skin squamous-cell carcinoma [244], PPARγ was found to be less expressed in highly vascularized high grade glioma than in low grade glioma [256]. Most in vivo [69,257,258,259,260,261,262,263,264] studies using different PPARγ agonists observed an inhibition of tumor angiogenesis upon PPARγ activation. In line with these findings, and suggesting that PPARγ activation inhibits tumor vascularization, the deletion of PPARγ in the mammary epithelium of an in vivo model of basal breast cancer lead to increased tumor vessel formation [265]. However, a recent study revealed that activated PPARγ promotes tumor vascularization and growth in breast cancer. Conformational changes in PPARγ induced by ligand activation provoked enhanced angiogenesis and faster tumor growth of mammary tumor cells [266]. A recent study further demonstrated that PPARγ agonists can enhance a pro-tumorigenic secretome in cancer cells, leading to increased tumor angiogenesis and progression [267].

**Figure 3:**
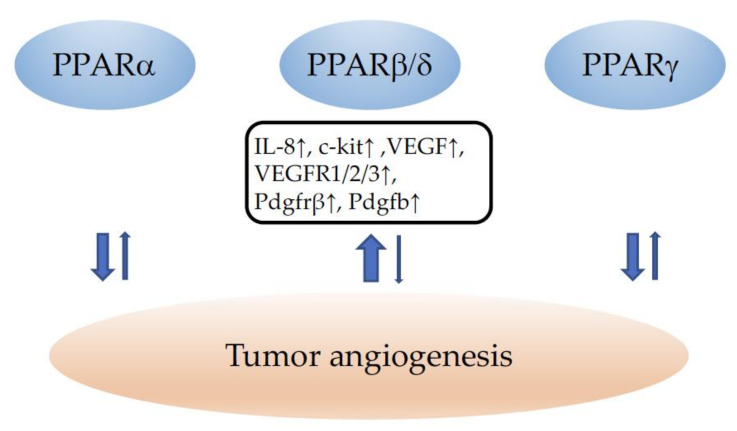
Summary of the influence of PPARα, PPARβ/δ, and PPARγ on tumor angiogenesis. ↓ indicates inhibition and ↑ an increase in angiogenesis. The width of the arrows corresponds to the number of studies reporting similar effects. IL-8: interleukin-8; c-kit: tyrosine-protein kinase Kit; VEGF: vascular endothelial growth factor; VEGFR1/2/3: vascular endothelial growth factor receptors 1/2/3; Pdgfrβ: platelet-derived growth factor receptor beta; Pdgfb: platelet-derived growth factor beta.

In conclusion, although PPARα and PPARγ seem to decrease tumor angiogenesis, caution should be taken regarding the therapeutical use of any PPAR agonist in the setting of susceptibility to cancer. The example of PPARβ/δ agonists which had been in clinical trials for the treatment of hyperlipidemia and cardiovascular diseases at the beginning of 2000 and turned out to provoke cancers in mice and rats after prolonged treatment, which put a stop to phase 4 trials [268], clearly illustrates the necessity of considering the therapeutic modulation of any PPAR with great care. Regrettably, in clinical studies investigating the use of PPAR modulation in cancer, the effects on tumor vascularization have not been evaluated (reviewed in [177]). A schematic summary of the role of PPARs in tumor angiogenesis is provided in Figure 3.

## 5. PPARs and Tumor Suppressors

### 5.1. PPARα

In addition to the positive regulation of growth-promoting signals, cancer progression is also characterized by the escape of tumor-suppressor action [32]. P53 has been shown recently to transcriptionally inhibit PPARα expression, which has been related to telomere dysfunction and aging, but a potential role in carcinogenesis remained unexplored [269]. On the contrary, PPARα binds to the p300 promoter, which results in the activation of the gene followed by the acetylation and stabilization of p53 in hepatocellular carcinoma [270]. The peroxisomal enzyme Acyl-CoA oxidase 2 (ACOX2) has been postulated as a tumor suppressor in hepatocellular carcinoma via the positive regulation of PPARα. Besides the upregulation of PPARα in hepatoma cell lines with ACOX2 overexpression, no mechanistic link between the two proteins has been explored [271]. Tribbles homolog 3 (TRIB3) has been identified as an oncoprotein in acute myeloid leukemia via the inhibition of apoptosis and autophagy. Mechanistically, this is due to the protein–protein interaction of TRIB3 with PPARα favoring the ubiquitination and degradation of PPARα; on the contrary, the pharmacological activation of PPARα promotes apoptosis and autophagy of leukemia cells [272]. PPARα expression was low in mouse and human colon cancers. The deletion of PPARα in mice reduced the expression of the retinoblastoma protein, resulting in increased expression of the methyltransferases DNMT1 and PRMT6 and, consequently, DNA and histone methylation and lower expression of the tumor suppressors p21 and p27 [273]. P21 seems to act upstream of PPARα under fasting conditions [274]. The tumor suppressor P63 represses PPARα in human keratinocytes [275]. The exact molecular regulation and consequences for tumor growth remained, in both reports, unexplored. PPARα transcriptionally activates the cell-cycle regulator p16Ink4a via a PPAR-response element and an SP1-binding site, and inhibits smooth-muscle cell proliferation, which is relevant to the prevention of intimal hyperplasia in cardiovascular disease [276]. Given the importance of p16Ink4a for cancer [277], potential relevance to tumor growth is likely. Fenofibrate treatment induced the expression of the thioredoxin-binding protein (TXNIP) tumor suppressor in neuroblastoma cells and induced apoptosis. As the inhibition of PPARα did not modify these results, it is likely that fenofibrate had a PPARα-independent effect [59] as was also shown in hepatocellular carcinoma cells [278]. N-Acetyl-Cysteine (NAC) has been described as a PPARα agonist, which inhibits the proliferation of non-small-cell lung carcinoma cells through the induction of p53 and the inhibition of p65, collaboratively reducing PDK1 promoter activity and expression [279]. PPARα activation supports the binding of HIF-1α to the von Hippel–Lindau tumor suppressor, thereby inducing HIF-1α degradation through the ubiquitin–proteasome pathway. Consequently, less Vegf is produced from cancer cells, and angiogenesis and tumor growth might be reduced [280].

Menin, the product of the MEN1 (multiple endocrine neoplasia type 1) tumor-suppressor gene was shown to physically interact with the PPARα protein to control the expression of genes involved in fatty-acid oxidation. The authors investigated a model of hepatic steatosis. Whether this interaction is relevant for tumorigenesis was not analyzed [281].

### 5.2. PPARβ/δ

We have reviewed the knowledge of PPARβ/δ and tumor suppressors before [38]. Recently, it has been shown that pancreatic intraepithelial neoplasias, which mostly harbor oncogenic KRAS mutations, are characterized by the upregulation of PPARβ/δ. PPARβ/δ stimulation via a high-fat diet, or when a specific agonist promotes tumor progression to pancreatic ductal adenocarcinoma [282]. Mechanistically, this is due to the activation of the CCL2/CCR2 axis in pancreatic epithelial cells, which induces an immunosuppressive tumor microenvironment [283]. The increased expression and activity of PPARβ/δ in K-Ras-transformed intestinal epithelial cells has already been described [284]. In hepatocellular carcinoma, SIRT4 acts as a tumor suppressor via the inhibition of PPARβ/δ-induced fatty-acid oxidation and the polarization of macrophages to a pro-inflammatory M1 phenotype [285]. The overexpression of PPARβ/δ in melanoma compared to normal skin has been reported in humans, mice, and horses [102,286]. The expression of PPARβ/δ was inversely correlated with the Wilms tumor suppressor WT1 [286], which is mostly considered as an oncogene [31,103,287,288,289,290,291,292,293]. PPARβ/δ activation inhibits melanoma-cell proliferation via the direct repression of WT1 [102], while WT1 stimulates melanoma-cell proliferation [103].

In smooth-muscle cells, the PPARβ/δ agonist L-165041 repressed the phosphorylation of the retinoblastoma protein pRB, and consequently, inhibited proliferation [294]. Whether a similar mechanism is acting in cancer cells is unknown. PPARβ/δ activation with GW0742 reduced SOX2 expression in neuroblastoma cell lines and induced cell differentiation, independently of the p53 status of the cells. Nevertheless, the authors concluded that PPARβ/δ induces neuroblastoma cell differentiation through the SOX2- and p53-dependent pathways [89].

The adenomatous polyposis coli (APC) tumor suppressor is frequently mutated in colon cancer and mouse models, and APC mutations are widely used in colon cancer research. Early reports showed that APC indirectly inhibits PPARβ/δ expression in colon cancer via the suppression of β-catenin/Tcf-4-mediated transcription [196]. The treatment of APCmin mice with the PPARβ/δ agonist GW501516 resulted in an increase in the number and size of intestinal polyps [77]. APC and axin tumor-suppressor-inactivating and β-catenin/Tcf-activating mutations are frequent in different types of cancers. Nearly 50% of ovarian endometrioid adenocarcinomas showed mutations with the dysregulation of β-catenin, which results in the upregulation of PPARβ/δ, MMP-7, Cyclin D1, Connexin 43, and ITF2 [295]. The overexpression of the tumor suppressor called transducer of ErbB-2.1 (Tob1) in gastric cancer cell lines reduced the expression and transcriptional activity of β-catenin, and consequently, of PPARβ/δ [296], supporting the regulation of PPARβ/δ by β-catenin in different cancer types. In breast cancer cells, PPARβ/δ activity seems to be tightly regulated via fatty-acid-binding protein 5 (FABP5). FABP5 binds natural ligands for PPARβ/δ and shuttles them to this nuclear receptor as a pre-requisite for activation. FABP5 expression is positively regulated via EGFR/ERK/phophatidylinositol-3-kinase signaling and activation of the transcription factor NF-kappaB, which is pro-tumorigenic in breast cancer, while Krüppel-like factor KLF2 inhibits FABP5 expression, reducing PPARβ/δ activity, and consequently, is tumor-suppressive [297].

### 5.3. PPARγ

The tumor suppressor Cyld has been proposed as a transcriptional target gene of PPARγ in mammary epithelial cells. Troglitazone stimulated Cyld mRNA expression and the activity of luciferase reporter/promoter constructs. Thereby, Cyld could act as a mediator of PPARγ-dependent anti-inflammatory and anti-proliferative activity in mammary epithelial cells [298]. The Wnt7a/Frizzled9/Gα16 pathway activates PPARγ to inhibit cell proliferation in non-small-cell lung cancer [299]. The retinoblastoma tumor-suppressor (Rb) protein interacts with E2F to suppress PPARγ expression. Consequently, in mice with compound loss of p53 and pRb, the tumor spectrum shifted from osteosarcoma (bone tumor) to hibernomas (brown-fat tumor), supporting the involvement of PPARγ in the cell-fate switch from bone- to adipose-tissue tumors [300]. The retinoic acid-producing enzyme aldehyde dehydrogenase 1a1 acts as a tumor suppressor in splenic B-cell subpopulations by regulating retinoic acid receptor alpha, zinc finger protein Zfp423, and PPARγ. The regulation of PPARγ was specific only to an IgG1(+)/CD19(+) cell population [301]. In hepatocellular carcinoma cells, PPARγ activation using rosiglitazone, or its overexpression, induced Cited2, which was associated with reduced cell growth and the induction of p15, p21, and p27. Chromatin immunoprecipitation confirmed that the binding of PPARγ to the Cited2 promoter sequence was direct [302]. Additionally, in bladder cancer cells, troglitazone increased the expression of p21 and p16Ink4a [217]. CCAAT/enhancer-binding protein-alpha (C/EBP-alpha) overexpression induced PPARγ expression, and secondary PPARγ directly activated p53 and induced apoptosis in rat hepatic stellate cells [303]. As C/EBP-alpha activating mutations are found in acute myeloid leukemia patients [304], this regulatory pathway might be relevant for cancer. In breast cancers, C/EBP-alpha shows low expression compared to its normal nuclear expression in ductal cells. Additionally, in this case, the overexpression of C/EBP-alpha was associated with increased PPARγ and p21 expression [305].

**Figure 4:**
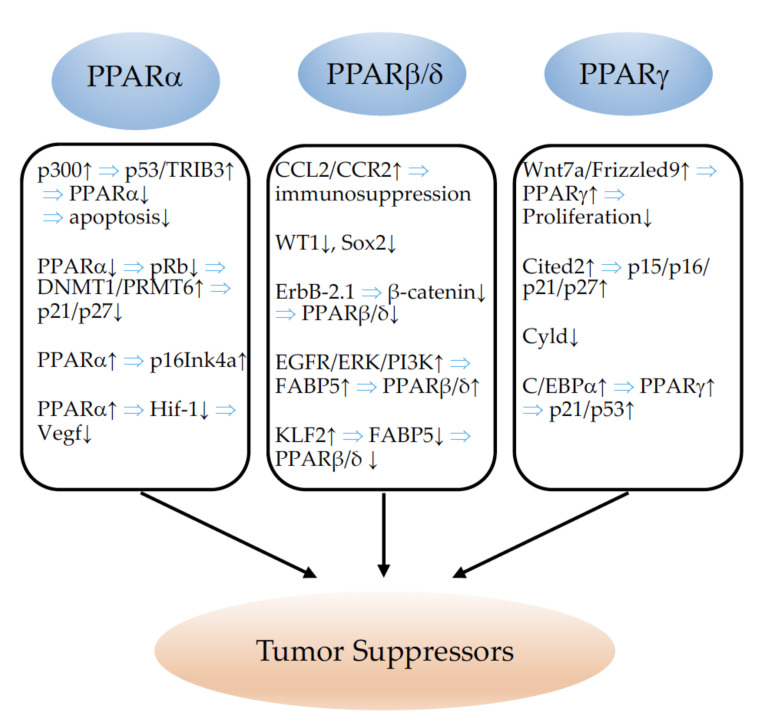
Illustration of the relationships between PPARα, PPARβ/δ, and PPARγ and tumor suppressors. ↓ indicates inhibition and ↑ indicates an increase. ⇒: leads to; p300: P300 transcriptional co-activator protein; p53: tumor protein p53; Trib3: Tribbles homolog 3; pRb: phosphorylated retinoblastoma protein; DNMT1: DNA (cytosine-5)-methyltransferase 1; PRMT6: protein arginine N-methyltransferase 6; p16Ink4a/21/27: tumor suppressors p16Ink4a, p21, p27; Hif-1: hypoxia-inducible factor-1; Vegf: vascular endothelial growth factor; CCL2: monocyte chemotactic protein-1; CCR2: receptor for monocyte chemoattractant protein-1; WT1: Wilms tumor 1 protein; SOX2: SRY-box transcription factor 2; ErbB-2.1: Erb-B2 receptor tyrosine kinase 2; EGFR: epidermal growth factor receptor; ERK: extracellular signal-regulated kinase; PI3K: phosphoinositide 3-kinase; FABP5: fatty-acid-binding protein 5; KLF2: Krüppel-like Factor 2; Wnt7a: Wnt family member 7A; Cited2: Cbp/p300-interacting transactivator 2; Cyld: cyld lysine 63 deubiquitinase; C/EBPa: CCAAT/enhancer-binding protein alpha.

Estrogen receptor alpha (ERα) interacts physically with PPARγ, and both proteins compete for the chance to bind to PPREs. While PPARγ activates transcription from this element, ERα represses transactivation. Thus, both proteins differentially modulate the proliferation of breast cancer cell lines in vitro [306]. The relationships between the different PPARs and tumor suppressors are schematically summarized in Figure 4.

## 6. PPARs in Invasion and Metastasis

### 6.1. PPARα

PPARα ligands were shown to inhibit the phorbol-ester-induced upregulation of Cox-2 and VEGF expression, both implicated in metastasis promotion, in a colon cancer cell line [307]. Similarly, PPARα ligands inhibited the transforming growth factor (TGF) α-induced expression of matrix metalloproteinase 9 (MMP 9), also strongly implicated in metastasis advancement [308]. Fenofibrate reduced the metastatic potential of melanoma cells in vitro and in vivo, implicating the downregulation of Akt phosphorylation [309,310]. The ligand activation of PPARα inhibited the formation of proangiogenic epoxyeicosatrienoic acids (EET) by the cytochrome P450 arachidonic acid epoxygenases (Cyp2c), and thereby reduced NSCLC growth and metastatic progression in vivo [65,247]. Acyl-CoA oxidase 2 (ACOX2) has been proposed to inhibit tumor progression and the metastasis of HCC trough a PPARα-dependent pathway [271]. In contrast, an elegant in vitro and in vivo study evidenced that PPARα favored metastasis. PPARα is required for the generation of immunosuppressive regulatory B cells, designated tBregs from B cells, which is induced by metabolites of the 5-lipoxygenase pathway. A deficiency of PPARα in B cells blocked the generation of tBregs, and thus, abrogated lung metastasis in mice with established breast cancer [311]. The metastasis of tumors to lymph nodes predicts disease progression and influences therapeutic schemes. Comparative metabolomic and transcriptomic analyses of primary tumors which had metastasized to lymph nodes demonstrated that metastasizing tumor cells undergo a metabolic shift towards fatty-acid oxidation (FAO). Most upregulated gene sets in the metastatic lymph node tumors were related to aspects of lipid biology, fatty-acid metabolism, and PPARα signaling pathways. The authors demonstrated that the activation of the transcriptional coactivator yes-associated protein (Yap) in lymph node metastatic tumors induced the upregulation of genes implicated in FAO. The inducible knockdown of Yap or of the inhibition of FAO suppressed lymph node metastasis [312]. Chen and coworkers reported that mitochondrial 3-hydroxy-3-methylglutaryl-CoA synthase (HMGCS2) enhanced the motility and metastasis formation of CRC and oral squamous-cell carcinoma (OSCC) cells in vitro and in vivo. This oncogenic function was found to be mediated through the direct binding of HMGCS2 to PPARα, which, in turn, led to the transcriptional activation of the proto-oncogene tyrosine-protein kinase Src, a target of PPARα. *HMGCS2* mRNA expression was further found to be associated with poor clinical prognoses and outcomes in patients [313]. It is highly interesting that the plasticizer di(2-ethylhexyl) phthalate (DEHP) and its hydrolysate mono(2-ethylhexyl) phthalate (MEHP) are major toxicants from plastics; nevertheless, a potential carcinogenic effect has not been investigated. Leng and colleagues demonstrated that MEHP treatment promoted the phosphorylation of Akt and the degradation of IκB-α, thus activating NF-κB and enhancing NF-κB nuclear translocation, which enhanced metastasis formation of ovarian cancer xenografts. The inhibition of PPARα by the antagonist GW6471 abrogated metastasis in vivo, indicating that the MEHP promotion of metastasis is mediated in a PPARα-dependent manner through the PI3K/Akt/NF-κB pathway [314]. In conclusion, PPARα favored metastasis in many model systems, also through its wider implication in metabolic and immunological processes. PPARα modulation is therefore, nowadays, not considered as a safe therapeutic option in the setting of cancer.

### 6.2. PPARβ/δ

The role of PPARβ/δ for the invasion and metastasis of cancers has recently been thoroughly reviewed in [38]. In a very detailed study, Abdollahi and colleagues demonstrated that PPARβ/δ expression levels were correlated with a higher malignant grade and distant metastasis formation in cancer patients with prostate, breast, and endometrial adenocarcinoma [249]. Additionally, in colorectal cancer, high expression of PPARβ/δ coincided with a high risk of developing distant liver metastases [71]. In contrast, in vitro studies using the PPARβ/δ agonist GW501516 in pancreatic [315] or breast cancer cells [316] reported decreased invasion capabilities of the tumor cells upon PPARβ/δ activation. A metastasis-inhibiting role of PPARβ/δ has been proposed by Lim and coworkers, who reported that treatment with the PPARβ/δ antagonist for 10 h increased melanoma cell migration and invasion. This antagonist had, so far, not been used in other studies, and the results were not confirmed by employing well-established antagonists such as GSK0660 or GSK3787 [317]. One group observed the downregulation of N-Cadherin upon PPARβ/δ agonist activation in a bladder cancer cell line, which has been suggested to diminish metastatic potential [318]. Most of the studies, however, confirm the invasion- and metastasis-promoting effects of PPARβ/δ, which were first suggested via analyses of PPARβ/δ expression in published large-scale microarray data from cancer patients [71,249]. A study by Zuo and colleagues identified several pro-metastatic genes as PPARβ/δ targets through the analysis of transcriptome profiling of HCT116 colon cancer cells, with or without the genetic deletion of PPARβ/δ. Using several experimental in vivo models (syngenic and orthotopic tumor inductions, different tumor-cell types), the authors showed that PPARβ/δ knockdown in cancer cells inhibited metastasis formation. The treatment of mice with the PPARβ/δ agonist GW0742 enhanced metastasis formation. It was further demonstrated that high expression of PPARβ/δ in cancer cells is the most important factor for metastasis formation as heterozygous PPARβ/δ mice developed fewer metastases than their wildtype littermates; however, the most important metastasis inhibition was observed when PPARβ/δ was deleted in cancer cells used for syngenic tumor induction. High PPARβ/δ expression in cancer cells additionally promoted tumor angiogenesis through increases in VEGF and IL-8. Finally, analyses of independent datasets from cancer patients (liposarcoma, colon, breast, and lung cancer) demonstrated that PPARβ/δ expression in cancer cells strongly influenced metastasis-free survival [252]. Our group confirmed the pro-metastatic effects of PPARβ/δ activation in vivo. PPARβ/δ agonist GW0742-treated animals with syngenic induced LLC1 tumors had significantly increased spontaneous lung and liver metastasis formation compared to controls injected with a vehicle. We further evidenced that the conditional inducible overexpression of PPARβ/δ in vascular cells was sufficient to promote metastasis formation [11]. High-fat diets are associated with carcinogenesis [319]; however, the underlying mechanisms are not well-understood. A recent study demonstrated the implication of PPARβ/δ in the pro-metastatic effects of dietary fats in colorectal cancer. The authors showed, first, that the activation of PPARβ/δ by GW501516 induced the expansion of colonic cancer stem cells (CSC) and boosted metastasis formation in vivo through the induction of the self-renewal regulatory factor Nanog. The activation of PPARβ/δ increased, whereas the knockout of PPARβ/δ decreased Nanog expression, and knockdown of Nanog abolished the metastasis-promoting effects of PPARβ/δ. Finally, the authors demonstrated that a high-fat diet mimicked the effects of PPARβ/δ activation by inducing Nanog, accelerating tumor formation, and increasing liver metastasis development. The knockout of PPARβ/δ inhibited the high-fat-diet-induced effects on tumorigenesis and progression [320]. Although few studies reported decreased metastasis-related events upon PPARβ/δ activation in vitro, the role of PPARβ/δ on metastasis remains to be defined in representative in vivo models, which unequivocally confirms the pro-metastatic functions of PPARβ/δ.

### 6.3. PPARγ

**Figure 5:**
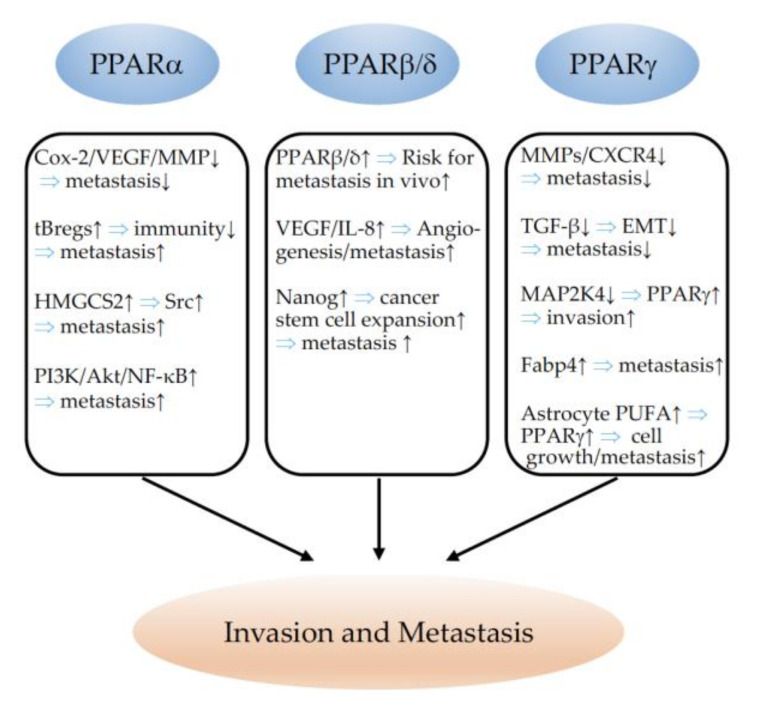
Schematic representation of the effects of PPARα, PPARβ/δ, and PPARγ on invasion and metastasis formation. ↓ indicates inhibition and ↑ indicates an increase. ⇒: leads to; Cox-2: cyclooxygenase-2; VEGF: vascular endothelial growth factor; MMP: matrix metalloproteinase; tBregs: immunosuppressive regulatory B cells; HMGCS2: 3-hydroxy-3-methylglutaryl-CoA synthase 2 (mitochondrial); Src: proto-oncogene tyrosine-protein kinase Src; PI3K: phosphatidylinositol 3-kinase; Akt: AKT serine/threonine kinase; NF-κB: nuclear factor kappa-light-chain-enhancer of activated B cells; IL-8: interleukin 8; CXCR4: C-X-C chemokine receptor type 4; TGF-β: transforming growth factor beta; EMT: epithelial–mesenchymal transition; MAP2K4: dual-specificity mitogen-activated protein kinase kinase 4; Fabp4: fatty-acid-binding protein 4; PUFA: polyunsaturated fatty acid.

Thiazolidinediones were found to inhibit the synthesis of matrix metalloproteinases (MMPs) and adhesion to the extracellular matrix (ECM) proteins of colon cancer cell lines [321], and to abolish lymph node and lung metastases in colon cancer xenograft models [322]. Similarly, linoleic acids have been reported to inhibit colon cancer metastasis through PPARγ activation [323]. Later, the downregulation of the chemokine receptor CXCR4 was further attributed to the metastasis-preventing effects of PPARγ in colon [324,325] as well as in breast cancer [326]. In line with these findings, low levels of PPARγ in colon cancers of patients were correlated with enhanced metastatic potential [327]. NSAIDs were reported to have beneficial effects on colon metastasis inhibition through their suppression of cancer stem cells, mediated through the suppression of Cox-2 and the activation of PPARγ [328]. Mammary tumors were found to metastasize less upon PPARγ activation due to decreased MMP production [329]. 15d-PGJ2 has further been shown to inhibit osteolytic breast cancer bone metastasis [330]. Additionally, NSCLC cells overexpressing PPARγ exhibited decreased metastatic potential [331]. A good study showed that the activation of PPARγ inhibited transforming growth factor β (TGF-β)-induced epithelial mesenchymal transition (EMT) in lung cancer cells. PPARγ-antagonized TGF-β–caused a loss of E-cadherin expression and inhibited the induction of mesenchymal markers and MMPs, thus preventing migration, invasion, and metastasis formation [332]. Rosiglitazone was found to suppress metastatic potential in gastric cancer, and the enhanced activity of PPARγ resulted in increased direct transcriptional activation of cellular adhesion molecule 3, which inhibits the migration and invasion of gastric cancer cells [333,334]. Modulation of the plasminogen activator system has been proposed to be one metastasis inhibiting mechanism of PPARγ activation in pancreatic cancer [335]. In hepatocellular carcinoma (HCC), low PPARγ expression was correlated with more advanced TNM (tumor, node, metastasis) stages [335], and PPARγ activation decreased the invasive and metastatic potential of cancer cells in vitro and in vivo through the downregulation of MMP9 and 13, and the upregulation of the extracellular matrix-regulator tissue inhibitors of metalloproteinase (TIMP) 3, E-cadherin, and spleen tyrosine kinase [336]. The high expression of Micro RNA 130b (miR-130b) in HCC was correlated with enhanced metastasis and the downregulation of PPARγ. Lowering miR-130b resulted in increased PPARγ expression and suppressed EMT in HCC cells [337]. An elegant study determined that PPARγ is required for the peroxisome proliferator-activated receptor-gamma coactivator-1α (PGC1α)-mediated inhibition of HCC metastasis. PGC1α inhibits the aerobic glycolysis of cancer cells through PPARγ-dependent inhibition of the WNT/β-catenin pathway [338]. However, an in vitro study suggested that PPARγ antagonists inhibited metastasis through the cleavage of vimentin in hepatocellular carcinoma [339]. Like the situation in HCC, microRNA 27b (miR-27b) has been suggested to downregulate PPARγ, and thereby, to promote the invasion of cervical carcinoma [340]. In squamous-cell carcinoma, the inhibition of PPARγ was proposed to decrease cell adhesion through the downregulation of integrin alpha 5 [238]. Later, doubts regarding the suggested beneficial effects of PPARγ activation for metastasis inhibition in lung cancer arose. Ahn and coworkers identified mitogen-activated protein kinase kinase 4 (MAP2K4) as a tumor suppressor in lung adenocarcinoma. MAP2K4 inhibited lung cancer cell invasion through the repression of PPARγ. MAP2K4 deficiency increased PPARγ expression and promoted cancer cell invasion, which could be reversed via PPARγ inhibition [341]. PPARγ agonist activation in orthotopic and spontaneous murine lung cancer models significantly increased metastasis formation through its upregulated expression in macrophages, which contributed to tumor progression and metastasis through increased arginase 1 expression. The inducible conditional knockout of PPARγ solely in macrophages reconstituted the beneficial roles of PPARγ ligand activation in lung cancer cell growth and metastasis inhibition [342]. The increased production of transforming growth factor β 1 (TGFβ1) in macrophages upon stimulation of PPARγ has been proposed as the underlying mechanism for the promotion of invasion and metastasis in this context [343]. Similarly, bone marrow adipocytes promote bone metastasis formation in prostate cancer, which is, in part, mediated through the PPARγ-induced activation of fatty-acid-binding protein 4 (Fabp4) [344]. Liliane Michaliks’ group further showed that the PPARγ agonist rosiglitazone activates a tumorigenic secretion program of cytokines, chemokines, and pro-angiogenic factors in melanoma cells, leading to a tumor progression- and metastasis-favoring microenvironment [267]. This, again, suggests that PPARγ may have anti-tumorigenic effects on cancer cells, but pro-tumorigenic effects on cells of the microenvironment, as was already described in the context of breast cancer [166]. The situation might be even more complex as truncated isoforms of PPARγ might further fuel the metastasis-promoting actions of tumor stromal cells. Niu and colleagues demonstrated that caspase-1 cleaves PPARγ, leading to a truncated isoform which translocates to mitochondria, resulting in the inhibition of medium-chain acyl-CoA dehydrogenase (MCAD) and fatty-acid oxidation. Thus, the differentiation of tumor- and metastasis-promoting macrophages is enhanced by the accumulation of lipid droplets [345]. Tumor-associated macrophages can be divided in two subgroups: M1 macrophages, which are pro-inflammatory cells involved in killing tumor cells, and M2 macrophages, which mediate tumor progression and metastasis. Shu and colleagues revealed the important role of integrin *β*3 in macrophage M2 polarization. The inhibition of integrin β3 blocked M2 polarization only in the setting of high PPARγ expression and activity, which indicates that the action of integrin β3 depends on PPARγ [346]. An excellent study unveiled the mechanism by which PPARγ facilitates brain metastasis formation from primary cancers: astrocytes, brain glial cells, have a high content of polyunsaturated fatty acids, which function as donors of PPARγ activation in invading cancer cells, thus enhancing proliferation and metastatic outgrowth to the brain. PPARγ expression was significantly higher in brain metastatic lesions than in the primary tumors of breast cancer and melanoma patients. PPARγ antagonist treatment reduced melanoma or breast cancer brain metastasis burden in animals. This further adds to the complexity regarding the role of PPARγ in cancer, which depends on the stage of cancer development. PPARγ might inhibit early primary cancer growth, but fuels advanced-stage metastatic formation [347]. The situation also becomes more complicated, as in several different tumor types such as prostate [348,349,350], bladder [351], pancreatic cancer [352], and myxoid liposarcoma [353], high levels of PPARγ expression in tumor cells are correlated with enhanced metastasis formation; this also indicates that a general beneficial effect of PPARγ expression in tumor cells on metastasis inhibition cannot be concluded. The major effects of PPARs for invasion and metastasis formation are illustrated in Figure 5.

## 7. PPARs and Replicative Immortality

### 7.1. PPARα

Stem cells in the intestinal epithelium lose their self-renewal capacity with aging due to decreased Wnt signaling. Mechanistically, high mTORC1 activity inhibits PPARα. In turn, Notum, a Wnt inhibitor, becomes activated via a lack of PPARα, and stem cell self- is inhibited [354]. Whether this mechanism also operates in cancer stem cells remains to be determined. High PPARα expression has been described in glioma stem cells compared to fetal neuronal stem cells. The inhibition of PPARα expression induced the downregulation of stem cell markers c-Myc, Sox2, and nestin, and induced senescence. In contrast to control cells with intact PPARα expression, knockdown cells did not form tumors in vivo, suggesting PPARα inhibition as a potential target for the inhibition of glioblastoma growth [60]. In line with this, the positive transcriptional regulation of CPT1C by PPARα was shown to inhibit senescence in different cancer cell lines in vitro [61]. Whether the shortened lifespan, hepatocarcinogenesis, and age-related lesions in the heart, kidney, and liver of PPARα-knockout mice reported earlier [355] are due to modifications in senescence remains unexplored; however, it seems more likely that alterations in apoptotic pathways are responsible for these phenotypes [356].

### 7.2. PPARβ/δ

The role of PPARβ/δ in replicative immortality, senescence, and cancer stemness was reviewed recently [38]. The pharmacological activation of PPARβ/δ inhibited senescence in human vascular smooth-muscle cells, coronary artery endothelial cells, keratinocytes, and cardiomyocytes [357,358,359,360]. On the contrary, higher PPARβ/δ expression was correlated with increased senescence in benign neurofibromas and colon adenomas [361], and senescence, in this case, was correlated with endoplasmic reticulum stress [362], which seems unusual. In endothelial cells, the lipid peroxidation product 4-HNE activated PPARβ/δ, resulting in the induction of thioredoxin-interacting protein (TXNIP) expression and senescence [363].

PPARβ/δ activation keeps neuronal and colonic cancer stem cells in an proliferative, undifferentiated state via the induction of Sox2 and Nanog [320,364], which, in the case of colon cancer, contributes to metastasis formation in response to fatty-acid intake [320]. PPARβ/δ is expressed in gastric progenitor cells where it upregulates Ccl20 and Cxcl1, contributing to chronic inflammation and malignant transformation [80]. Furthermore, PPARβ/δ contributes to stemness through protein–protein binding with β-catenin and the transcriptional activation of low-density lipoprotein receptor-related protein 5 (LRP5), which acts as a Wnt co-receptor [365]. Whether this is the case in cancer stem cells is an open question. In general, it is currently difficult to conclude whether the PPARβ/δ-dependent induction/inhibition of senescence might promote or delay cancer progression, as senescence, on one hand, is a gatekeeper to prevent cancer, but on the other hand, it might also contribute to the initiation and progression of a second tumor [366,367,368,369].

### 7.3. PPARγ

**Figure 6:**
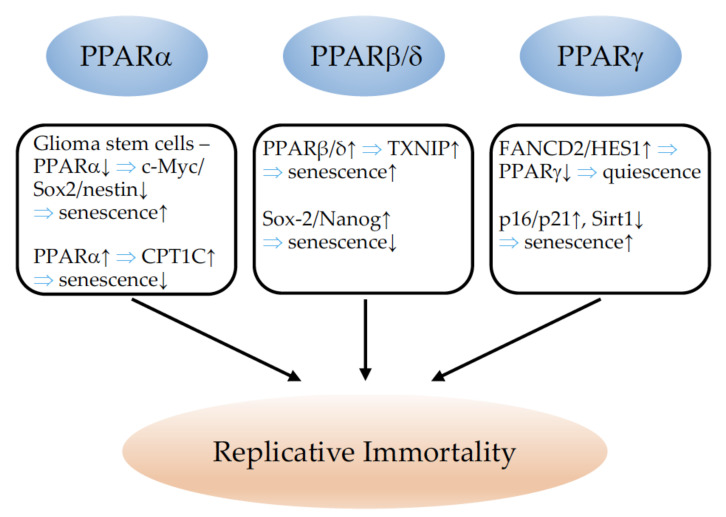
Summary of the effects of PPARα, PPARβ/δ, and PPARγ on senescence and replicative immortality. ↓ indicates inhibition and ↑ indicates an increase. ⇒: leads to; c-Myc: MYC proto-oncogene; Sox2: SRY (sex-determining region Y)-box 2; CPT1C: carnitine palmitoyltransferase 1C; TXNIP: thioredoxin-interacting protein; FANCD2: Fanconi anemia, complementation group D2; HES1: hes family bHLH transcription factor 1; Sirt1: sirtuin 1.

Recently, it was shown that the Fanconi anemia protein FANCD2 and Hairy Enhancer Split 1 (HES1) collaborate in the transcriptional repression of PPARγ to keep hematopoietic stem cells in a quiescent state and to avoid stem cell exhaustion, as well as hematological malignancies [370]. PPARγ is also required for enhanced glucose-stimulated insulin secretion in senescent pancreatic beta cells with aging [371]. Whether this affects cancer metabolism and growth is currently undetermined. PPARγ has different effects in stroma and cancer cells. PPARγ overexpression reduced breast cancer cell growth in xenograft models, and was associated with increased autophagy and the inhibition of angiogenesis; meanwhile, overexpression in stromal cells enhanced tumor growth, which has been related to the increased expression of autophagic markers, the production of lactate, cell hypertrophy, mitochondrial dysfunction, and senescence, as illustrated by higher p16/p21 expression and beta galactosidase [166]. In cell-culture models, PPARγ inhibits the expression of silent information regulator type 1 (SIRT1), a molecule known to delay senescence, which is in agreement with the senescence-promoting effects of PPARγ described above [372]. In human fibroblasts, PPARγ transcriptionally activates p16 and induces senescence [373]. In human colon cancer samples, a significant correlation between PPARγ and the expression of pRb, cyclin D1, p16, and p21 was found; however, surprisingly, PPARγ expression did not correlate with the stage, grade of differentiation, metastasis, tumor proliferative capacity, or patient survival [374]. Additionally, the opposite effect, involving the pioglitazone-induced induction of proliferation via the inhibition of P16 expression in adipocyte progenitors, has been described [375]. Pioglitazone treatment in mice activated telomerase and inhibited p16 expression and senescence in vascular cells [376]. The effects of PPARs on replicative immortality and senescence are summarized in Figure 6.

## 8. PPARs and Tumor Metabolism

PPARs are important mediators of lipid and glucose metabolism [1,377]. Glucose and fatty acids serve to sustain cancer-cell proliferation and fatty-acid function as signaling molecules and membrane components of cancer, as well as immune cells [32,378]. A major metabolic anomaly in cancers, i.e., the dependence on aerobic glycolysis for energy production, was described by Otto Warburg nearly 100 years ago [379]. Furthermore, as a general characteristic of cancer metabolism, the rapid growth of tumors results in hypoxia and the stabilization of hypoxia-inducible transcription factors (Hif) [380,381], which induce or repress the expression of downstream target genes, with relevance to cancer growth, e.g., VEGF [382], WT1 [383], PPARα [384], glucose transporters, and many others (reviewed in [385]). As the expression of different PPARs varies between cancer types, here, we will summarize the knowledge on PPARs in the metabolic regulation of distinct tumors.

### 8.1. PPARα

The hepatocarcinogenic effects of peroxisome proliferators in mice were already described in the 1970s [386]. PPARα activation induces the key genes of fatty-acid metabolism, which results in the increased generation of reactive oxygen species [387] and favors carcinogenesis. This predisposing role is modified by antioxidant defense mechanisms, age, and nutritional status (reviewed in [388]). Furthermore, interactions between different cell types modify the response to PPAR modulators.

Fibrates also favor oxidative metabolism in cytotoxic T cells. Fenofibrate reduced glucose’s utilization of cancer cells and stromal cells and shifted their metabolisms to fatty-acid use [389]. The glucose in the tumor environment was available for CD8 T cells and tumor infiltrating lymphocytes, which enhanced the success of tumor vaccination in a mouse model [390]. A potential use of PPAR ligands for the metabolic reprogramming of T cells in cancer immunotherapy has been described and reviewed before [391,392]. In a recent study, it was shown that the addition of fibrates to immune checkpoint inhibitors in patients with non-small-cell lung cancer increases overall survival, which was not the case in patients receiving chemotherapy [393]. Whether this effect is due to shifts in metabolism or involves other cancer hallmark capabilities is unknown. Nevertheless, it is an exciting finding linking PPAR research to clinical application.

Further crosstalk exists between adipocytes and tumor cells. Obese or diabetic patients are at increased risk of breast cancer [394,395]. The co-culture of adipocytes and breast cancer cell induces the expression of genes involved in inflammation and lipid metabolism (IL1, PLIN2, ANGPTL4). ANGPTL4 is a downstream target of PPARα. Consequently, the pharmacological inhibition of PPARα reduced ANGPTL4 expression, which is involved in adipose-tissue-induced β-oxidation, proliferation, and the invasion of breast cancer cells [396]. High glucose activated PPARα and PPARγ expression in breast cancer cell cultures [40]. Sirt6 activated PPARα expression, promoted beta-oxidation and mediated the PPARα-dependent inhibition of SREBP-dependent cholesterol and triglyceride synthesis in the livers of mice [397]. Whether this pathway is relevant for tumorigenesis remains to be determined. Activating mutations in the beta-catenin gene are frequently found in hepatocellular carcinomas. Beta-catenin acts as an activator of PPARα, which stimulates fatty-acid oxidation as the major metabolic pathway of beta-catenin-dependent hepatocellular carcinoma. Consequently, a knockout of PPARα and the inhibition of fatty-acid oxidation using the CPT-1 inhibitor etomoxir reduced hepatocellular carcinoma progression [398].

Aldehyde dehydrogenase (ALDH7A1) acts upstream of PPARα by providing metabolites which act as ligands for this receptor. The knockdown of ALDH7A1 increased cell migration and invasion. Low levels of the aldehyde dehydrogenase protein were correlated with poor clinical outcome in hepatic and kidney cancer patients [399]. The PPARα agonist Wy14,643 reduced Glucose transporter 1 (Glut1) expression, glucose transport, and the proliferation of different cell lines, suggesting anti-tumorigenic action in this model [400,401]. In contrast, PPARα is highly expressed in glioblastoma and glioma stem cells, and its inhibition results in the downregulation of key regulators of fatty-acid oxygenation, ACOX1 and CPT1A, and reduced tumor growth in mice [60]. Surprisingly, the inhibition of aerobic glycolysis, mitochondrial damage, and reduced glioblastoma growth in mice in response to fenofibrate treatment has also been described [402]. The PPARα antagonist GW6471 attenuated enhanced fatty-acid oxidation and oxidative phosphorylation, blocked enhanced glycolysis, and reduced tumor growth in a renal-cell carcinoma model in nude mice [194].

### 8.2. PPARβ/δ

PPARβ/δ function in cancer and metabolic alterations were previously investigated in colon cancer. The first publications were already controversial (reviewed in [38,403]). PPARβ/δ activation stimulates calcineurin expression [404], which induces Hif-1 stabilization [405]. Hypoxia, in turn, stimulates the transcriptional activation of PPARβ/δ in colon cancer cells via association with p300. PPARβ/δ deficiency in colon cancer cells reduces hypoxia-induced VEGF and IL6 expression, which links PPARβ/δ to tumor angiogenesis and immune response in colon cancer [406].

A mouse model of PPARβ/δ overexpression in gastric progenitor cells demonstrated the development of invasive gastric tumors in aging animals. Metabolic profiling revealed that these tumors do not require glycolysis but fatty-acid oxidation for tumor progression [407]. Additionally, a high-fat diet has been shown to induce fatty-acid oxidation depending on PPARβ/δ, which is associated with intestinal stem cell activation and enhanced tumorigenesis [408], as well as colorectal metastasis formation via the activation of Nanog in colonic cancer stem cells [320]. Epidemiological studies suggest a positive association of saturated fatty acids with colon cancer risk, while an inverse association exists for polyunsaturated fatty acids [409]. However, experimental studies showed that saturated long-chain fatty acids (SLCFAs) might inhibit the proliferation of some cancer cell lines, while unsaturated long-chain fatty acids (ULCFAs) induce cancer cell growth [410,411]. These differences could be related to the expression of fatty-acid-binding protein 5 (FABP5), retinoic acid receptors (RAR), and PPARβ/δ. Both SLCFAs and ULCFAs bind to FABP5, which dislodges retinoic acid and endogenous PPAR ligands from this transport protein. Depending on the presence of RARs, retinoic acid will bind to this receptor and activate it. SLCFAs reduce PPARβ/δ activity, while ULCFA/FABP5 complexes translocate to the nucleus where ULCFAs act as ligands for PPARβ/δ [412]. Consequently, a lack of FABP5 has been shown to inhibit mammary tumorigenesis [95]. These data are in general agreement with a pro-tumorigenic effect of PPARβ/δ, but point also to the complexity of different ligands, PPAR, RXR, and fatty-acid-binding protein expression in each individual tumor sample.

As an epigenetic mechanism, N1-methyladenosine methylation in tRNA via TRMT6/TRMT61A enhances PPARβ/δ translation, which augments cholesterol synthesis and Hedgehog signaling in liver cancer stem cells to support hepatic carcinogenesis [413]. The PPARβ/δ agonist GW501516 induced the expression of Glut1 (glucose transporter 1) and SLC1A5 (solute carrier family 1 member 5), which favors glucose and glutamine influx, thereby enhancing the proliferation of different cancer cell lines in vitro [78,414]. These effects were reversed by metformin. The molecular mechanisms were not investigated. In hepatocellular carcinoma resistant against the tyrosine kinase inhibitor sorafenib, a higher glutamine metabolism and reductive glutamine carboxylation dependent on PPARβ/δ were reported. The inhibition of PPARβ/δ reversed these metabolic alterations and sensitized the tumors to sorafenib, suggesting that sorafenib resistance in these tumors depends on PPARβ/δ-dependent metabolic alterations and might be treated with PPARβ/δ antagonists [415].

### 8.3. PPARγ

The role of PPARγ in metabolism and cancer has been reviewed before [228,416,417]. Part of the beneficial effects in cancer might simply be attributed to the reduction in tumor cachexia, which was associated with better survival in animal models [418,419]. The complex interactions between stroma and cancer cells are underlined by the observation that PPARγ activation in cancer cells reduces tumor growth, while overexpression in stromal cells enhances breast cancer growth in mice. In this model, cancer cells induce autophagy, glycolysis, and senescence in stromal cells, while stromal cells generate L-lactate, ketones, glutamine, amino acids, and fatty acids that are used by cancer cells to enhance their tumorigenic potential [166].

New data showed that interleukin-4 (IL-4) induces the expression of hematopoietic prostaglandin D2 synthase, thereby enhancing the endogenous levels of prostaglandin D2 and its metabolites. They act via PPARγ to reduce the severity of acute myeloid leukemia (AML) in mouse models and patient cells, suggesting IL-4 as a potential additional therapeutic option for AML [420]. Ubiquitin-specific protease 22 (USP22) stabilizes PPARγ due to deubiquitination, which increases acetyl-CoA carboxylase (ACC) and ATP citrate lyase (ACLY) expression and induces de novo lipogenesis as a risk factor for hepatocellular carcinoma (HCC). Consequently, PPARγ inhibition might reduce HCC progression [421]. In prostate cancer cells, PPARγ stimulates AKT serine/threonine kinase 3 (AKT3) expression, which favors PGC1α localization to the nucleus, mitochondrial biogenesis, and elevates ATP levels, ultimately leading to tumor-cell proliferation and metastasis via an enhanced epithelial–mesenchymal transition [350]. N-3 polyunsaturated fatty acids stimulated Syndecan 1 expression via PPARγ activation in prostate epithelium and prostate cancer cells [422]. The authors suggested chemo preventive properties of n-3 fatty acids in prostate cancer via this pathway, which was not proven experimentally. Additionally, in metastatic brain tumors, PPARγ is activated and contributes to metastatic spreading of the tumor cells due to the generation of lipid-derived endogenous PPAR activators from surrounding astrocytes [347].

Acyl-coenzyme-A-binding protein (ACBP) is a direct downstream effector of PPARγ that induces lipogenesis [423]. The long non-coding RNA MALAT1 acts upstream of PPARγ and might directly activate the PPARγ promoter to induce adipogenesis. Low expression of MALAT1 in cancer patients is associated with tumor cachexia and poor survival [424]. The esophageal adenocarcinoma-specific master regulator transcription factors (MRTFs) ELF3, KLF5, GATA6, and EHF activate PPARγ. PPARγ, in turn, enhances the synthesis of fatty acids, phospholipids, and sphingolipids and, in a positive feedback loop, induces MRTF expression, suggesting a pro-cancerogenic function in esophageal adenocarcinoma [425]. In metastatic prostate cancer, the situation seems comparable. PPARγ promotes the growth of this cancer type via the activation of lipid signaling pathways, i.e., the upregulation of fatty-acid synthase, acetyl-CoA carboxylase, and ATP citrate lyase. The inhibition of PPARγ reduces lipid synthesis and tumor growth [348]. Furthermore, PPARγ promotes prostate cancer growth via the induction of VEGF expression [426].

Hypoxia induces the stabilization of Hif-1α, which suppresses PPARγ in non-small-cell lung cancer (NSCLC). This is associated with uncoupling protein 2 (UCP2) downregulation, which results in the production of reactive oxygen species, upregulation of the ABC transporter protein ABCG2, elevated glucose uptake, and reduced oxygen consumption. These mechanisms might contribute to chemoresistance in NSCLC [427]. Whether PPARγ agonists sensitize NSCLCs to chemotherapy and are of therapeutic benefit, or whether other Hif-1α-dependent signaling pathways might interfere in this tumor type, could be relatively easily answered from researchers’ long clinical experience with the use of PPARγ agonists. Earlier studies found that PPARγ inhibits the growth and invasiveness of NSCLCs and other cell lines via the inhibition of Cox-2 expression [428] and the reduction in prostaglandin E(2) production [429,430].

**Figure 7:**
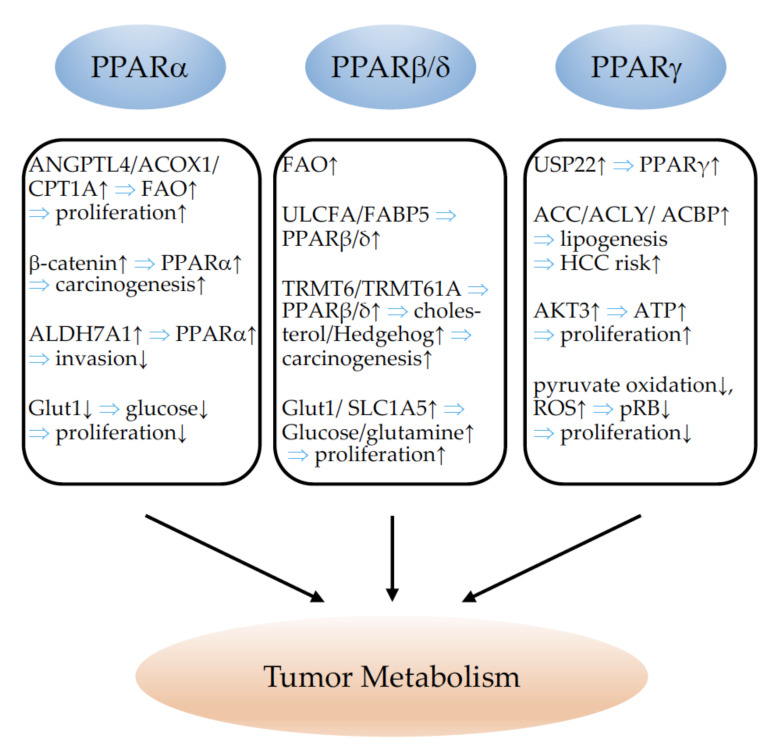
Summary of the effects of PPARα, PPARβ/δ, and PPARγ on tumor metabolism. ↓ indicates inhibition and ↑ indicates an increase. ⇒: leads to; ANGPTL4: angiopoietin-like 4; ACOX1: acyl-coenzyme A oxidase 1; CPT1A: carnitine palmitoyltransferase 1A; FAO: fatty-acid oxidation; ALDH7A1: aldehyde dehydrogenase 7 family member A1; Glut1: glucose transporter 1; ULCFA: unsaturated long-chain fatty acids; FABP5: fatty-acid-binding protein 5; TRMT6: tRNA methyltransferase 6 non-catalytic subunit; TRMT61A: tRNA methyltransferase 61A; SLC1A5: solute carrier family 1 member 5; USP22: ubiquitin-specific peptidase 22; ACC: acetyl-CoA carboxylase; ACLY: ATP citrate lyase; ACBP: acyl-CoA binding protein; HCC: hepatocellular carcinoma; AKT3: AKT serine/threonine kinase 3; ATP: adenosine triphosphate; ROS: reactive oxygen species; pRB: phosphorylated retinoblastoma protein.

A clinical trial of at least phase 2 in CML patients showed some beneficial effects of the addition of pioglitazone [156]. The PPARγ agonist pioglitazone was found to induce a metabolic switch that inhibits pyruvate oxidation, reduces glutathione levels, and increases reactive oxygen species (ROS) levels, inducing the hypo-phosphorylation of the retinoblastoma protein (RB) and cell-cycle arrest [173]. In a prostate cancer cell-derived tumor spheroid culture system, pioglitazone lowered the pH, decreased oxygen consumption, and increased lactate secretion. Other glitazones had similar effects [431]. Troglitazone and ciglitazone inhibited aerobic glycolysis, induced SIRT1 expression and endoplasmic reticulum stress in cancer cells, and induced autophagy and apoptosis independently of PPARγ [432]. Thus, it remains difficult to conclude specific PPARγ effects in cancer metabolism from studies using thiazolidinediones. The major effects of PPARs on tumor metabolism and the functional consequences are summarized in Figure 7.

## 9. PPARs and Cancer Immunity

### 9.1. PPARα

Over twenty years ago, a regulatory function of PPARα in inflammatory processes was already proposed. PPARα-null mice displayed a prolonged inflammatory response to stimulation with leukotriene B4, an activating ligand for PPARα [433]. PPARα has further been shown to be the predominant PPAR expressed by T and B lymphocytes. Following T-cell activation, PPARα was downregulated, whereas PPARγ expression increased [434]. PPARα is also already expressed in monocytes and upregulated during their maturation into macrophages. PPARα agonists induce the apoptosis of activated, but not of un-activated macrophages [435]. PPARα plays a major role in the immunomodulation caused by peroxisome proliferators (PPs). The group of J. W. DePierre demonstrated that several PPs, including perfluorooctanoic acid (PFOA), di(2-ethylhexyl)phthalate (DEHP), Wy-14 643, and nafenopin caused dramatic thymic and splenic atrophy in wildtype mice, with decreases in both, B- and T-cell populations, with the greatest reduction in the immature CD4^+^CD8^+^ population [436]. In contrast to wildtype animals, the authors did not observe these immunomodulatory effects of PPs in PPARα-knockout animals, identifying PPARα as the crucial regulator of PP-induced immunomodulation [437]. PPARα activation further decreases early B-cell development within the bone marrow [438]. The ability of PPs to suppress adaptive immunity in rodents may contribute to the development of hepatocarcinogenesis (reviewed in [439]) in response to these same substances. Using PPARα-deficient mice fed a high-fat diet, PPARα has been shown to protect against obesity-induced liver inflammation via the downregulation of inflammatory genes and the attenuation of adipose-tissue inflammation, partially through the prevention of fat accumulation in the liver [440]. Similarly, in a human-like hyperlipidemic mouse model (APOE2 knock-in mice) fed a western-type high-fat diet, fenofibrate treatment decreased hepatic macrophage accumulation, abolished steatosis, and reduced the expression of inflammatory genes [441]. Similarly, beneficial effects have been reported for PPARα activation in inflammatory bowel disease [442,443,444]. Michalik and colleagues evidenced the implication of PPARα in skin wound healing. They showed that PPARα is mainly involved in the initial inflammatory phase after injury, which precedes normal wound repair. PPARα-deficient mice exhibited a significant delay in the early-phase healing process, characterized by the impaired recruitment of neutrophils and monocytes/macrophages to the wound bed. This uncontrolled inflammation accounts for the transient delay of healing observed in PPARα-deficient animals [445]. The feeding of PPARα agonists to aged mice restored the cellular redox balance, evidenced by a lowering of tissue lipid peroxidation, an elimination of constitutively active NF-κB, and a loss of spontaneous inflammatory cytokine production [446]. PPARα further directly represses pro-inflammatory genes such as STAT, activator protein-1 (AP-1), NF-κB, and nuclear factor of activated T cells (NFAT) and activates anti-inflammatory components such as interleukin-1 receptor antagonist (IL1-Ra), Vanin-1, and mannose-binding lectin (MBL), as reviewed in [447,448]. PPARα further functions as a natural suppressor of the enzyme 11-β hydroxysteroid dehydrogenase 1 (HSD11B1), a widely expressed enzyme that converts biologically inactive cortisone to the functional glucocorticoid cortisol, known to exert multiple immunomodulatory effects [449]. In contrast to the suggested anti-inflammatory role of PPARα, Hill and colleagues observed, in a mouse model of endotoxemia, higher TNFα levels in animals treated with PPARα agonists [450]. Most studies have suggested a role for PPARα in the downregulation of endothelial cell (EC) inflammatory responses. PPARα agonists limited chronic inflammation mediated by VCAM-1 and monocytes without affecting acute inflammation mediated by E-selectin and neutrophil binding [451]. The PPARα agonist fenofibrate inhibits VCAM-1 transcription, in part, by inhibiting NF-κB [452]. The repression of NF-κB via PPARα activation was also identified as the mechanism for the inhibition of interleukin-6 and for the prostaglandin production and expression of COX-2 in human aortic smooth-muscle cells [453]. Lee and colleagues demonstrated a pro-inflammatory role of PPARα in the mediation of the activation of endothelial cells to produce monocyte chemotactic activity in response to oxidized phospholipids and lipoproteins [454]. Based on in vivo and in vitro studies, PPARα appears to have predominantly anti-inflammatory effects, although, in some studies, the pro-inflammatory consequences of PPARα activation have been demonstrated. Inflammation can either support or inhibit cancer growth. An outstanding report evidenced that PPARα-expressing granulocytes, mainly neutrophils, are required for tumor growth. PPARα deficiency in the host suppressed tumor growth via the induction of a plain inflammation capable of suppressing tumor angiogenesis, mainly through increased production of thrombospondin (TSP)-1 [53]. PPARα deficiency has further been demonstrated to inhibit tumor growth by impairing regulatory T-cell (Treg) functions and by supporting a pro-inflammatory Th1 T-cell phenotype [54]. These findings clearly support the negative impact of PPARα on the immune environment in the setting of cancer. However, from a metabolic point of view, PPARα activation could also be beneficial in reducing tumor growth. Tumor-infiltrating lymphocytes (TILs) suffer from the metabolic stress of hypoxia and hypoglycemia in the tumor environment. To preserve their effector functions, it has been demonstrated that they are able to enhance PPARα signaling and fatty-acid (FA) catabolism. Fenofibrate treatment further improved TILs’ ability to reduce tumor growth via the promotion of FA catabolism [455]. Nevertheless, a recent study evidenced that PPARα drives dendritic-cell immune dysfunction in cancer. Dendritic cells are key players in the initiation, programming, and regulation of anti-tumor responses. Fatty-acid-carrying tumor-derived exosomes (TDEs) activate PPARα, which, in turn, leads to excess lipid-droplet biogenesis and enhanced FAO, provoking a metabolic shift to mitochondrial oxidative phosphorylation and dendritic-cell immune dysfunction. The inhibition of PPARα reversed the TDE-induced immune dysfunction of dendritic cells and increased immunotherapy effectiveness [456]. Cancer development and its response to therapy are regulated by inflammation. PPARα is clearly involved in both chronic inflammation, facilitating tumor progression and treatment resistance, and acute inflammatory reactions, often leading to anti-tumor immune responses. Due to its plethora of immunomodulatory and metabolic effects, PPARα might either promote or suppress tumor progression, provoking opposing effects on therapeutic outcomes.

### 9.2. PPARβ/δ

The function of PPARβ/δ in immunomodulation has been extensively reviewed in [457] and [38]. The first attestations to a possible implication of PPARβ/δ in immune processes resulted from observations following skin injury. PPARβ/δ-deficient animals displayed a greater hyperplastic response in skin after O-tetradecanoylphorbol-13-acetate (TPA) treatment than wildtype controls and did not respond to NSAID sulindac treatment in contrast to their wildtype counterparts [458]. Tan and colleagues showed that the pro-inflammatory mediators TNF-α, interferon (IFN)-γ, and tissue plasminogen activator (TPA) upregulate PPARβ/δ expression in primary keratinocytes isolated from wildtype mice. The increase in PPARβ/δ strongly accelerated the differentiation of keratinocytes and increased their resistance to apoptotic signals, which was abolished in PPARβ/δ-deficient mice [459]. PPARβ/δ immune functions have frequently been studied in the setting of atherosclerosis. PPARβ/δ, highly expressed in endothelial cells [460], inhibits endothelial-cell inflammatory responses which lead to leukocyte recruitment [461,462,463,464]. In macrophages, PPARβ/δ controls inflammation through its association with the transcriptional co-repressor B-cell lymphoma (BCL)-6 which blocks the anti-inflammatory actions of BCL-6 and increases levels of inflammatory mediators such as methyl-accepting chemotaxis proteins (MCP)-1 and 3, and IL-1β. Following ligand binding to PPARβ/δ, BCL-6 is released and can repress inflammation [463]. The PPARβ/δ agonist GW0742 was shown to inhibit COX-2 and inducible nitric oxide synthase (iNOS) in macrophages [465]. PPARβ/δ has further been implicated in the switch of pro-inflammatory M1 macrophages to the anti-inflammatory M2 phenotype [466,467]. The PPARβ/δ agonist GW0742 strongly induced arginase I expression in macrophages, which impacted the balance of Th1/Th2 responses [468]. It is highly interesting that PPARβ/δ functions as a transcriptional basis for the detection and the discarding of apoptotic cells by macrophages, thus ensuring the timely and effective clearance of dying cells and increased anti-inflammatory cytokine production [469]. Adhikary and colleagues investigated the PPARβ/δ-regulated signaling network in human monocyte-derived macrophages. PPARβ/δ agonists inhibited the expression of multiple pro-inflammatory mediators and induced an anti-inflammatory phenotype. Of note, the authors also identified the immune stimulatory effects of PPARβ/δ agonists, which were reflected functionally by enhanced macrophage survival under hypoxic stress and stimulated CD8^+^ T-cell activation upon PPARβ/δ activation [470]. In ovarian cancer, tumor-associated ascites contains high concentrations of polyunsaturated fatty acids (PUFAs), which function as potent PPARβ/δ agonists in macrophages. They accumulate in lipid droplets in tumor-associated macrophages (TAMs), providing a reservoir of PPARβ/δ ligands, and induce the upregulation of PPARβ/δ target genes associated with immune regulation and tumor progression, such as CD300A, mitogen-activated protein kinase (MAP3K) 8 and angiopoietin-like 4 (ANGPTL4) [471]. Little is known about their expression and function in other immune cell types. PPARβ/δ expression has been described in lymphocytes [472] and has been suggested to stimulate T-cell proliferation and to inhibit INF-induced apoptosis [473]. Recently, the PPARβ/δ agonist GW501516 has been shown to enhance the efficacy of adoptive cell therapy by enhancing the expression of carnitine palmitoyl transferase 1A (CPT1A), the rate-limiting enzyme of FAO, in activated CD8^+^ T cells. Activated T cells produced more IFN and T-bet, which prevent cell exhaustion [474]. PPARβ/δ is further implicated in monocyte-to-dendritic cell maturation. Interestingly, PPARβ/δ agonists and naturally occurring ligands such as fatty acids drive the maturation of dendritic cells with an atypical phenotype, characterized by reduced expression of IL-10 and IL-12, and reduced stimulatory effects on leucocytes [475]. Mast cells, able to rapidly respond to modifications in their environment, favor tumor progression through the induction of angiogenesis and tissue remodeling (reviewed in [476]). Recently, it has been demonstrated that PPARβ/δ might be involved in mast-cell maturation and contribute to inflammatory responses in mast cells; however, the consequences of PPARβ/δ in mast cells in the context of cancer have not been studied [477]. Natural-killer (NK) cells have major functions in anti-tumor immunity, and obesity has been shown to reduce NK cell cytotoxic effector functions. Lipids induce metabolic defects, causing NK cell failure, leading to a loss of anticancer functions. NK cells express PPARα and PPARβ/δ, and agonists for both PPARs induce a dysfunctional NK cell phenotype; this mimics the NK cell phenotype in obesity, which is unable to exert anti-tumor functions [478]. In general, PPARβ/δ appears to be anti-inflammatory. However, the few studies investigating PPARβ/δ immune function in cancer describe pro-tumorigenic consequences such as the stimulation of tumor-promoting TAMs [471], and the inhibition of the cytotoxic anti-cancer effects of NK cells [478].

### 9.3. PPARγ

**Figure 8:**
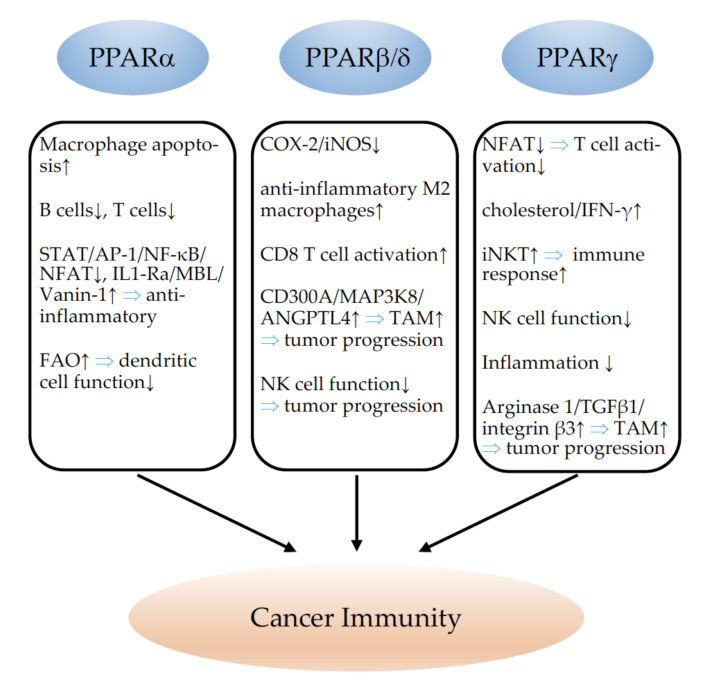
Effects of PPARα, PPARβ/δ, and PPARγ on cancer immunity. ↓ indicates inhibition and ↑ indicates an increase. ⇒: leads to; STAT: signal transducer and activator of transcription; AP-1: activator protein-1; NF-κB: nuclear factor kappa-light-polypeptide-gene-enhancer in B cells; NFAT: nuclear factor of activated T cells; IL1-Ra: interleukin 1 receptor antagonist; MBL: mannose-binding lectin; FAO: fatty-acid oxidation; COX-2: cyclooxygenase-2; iNOS: nitric oxide synthase 2 inducible; CD: cluster of differentiation; MAP3K8: mitogen-activated protein kinase kinase kinase 8; ANGPTL4: angiopoietin-like 4; TAM: tumor-associated macrophages; NK: natural-killer cell; IFN-γ: interferon gamma; iNKT: invariant natural-killer T cell; TGFβ1: transforming growth factor beta 1; integrin β3: integrin subunit beta 3.

PPARγ agonists mediate a direct inhibitory role in T-cell immune responses. They negatively regulate T-cell activation by inhibiting the nuclear factor of activated T cells (NFAT) and subsequent IL-2 production [479,480]. Consequently, the limitation of T-cell activation by PPARγ activation improves inflammatory diseases [481,482,483]. PPARγ activation has also been demonstrated to decrease T-cell proliferation through the induction of apoptosis [484]; however, other studies have shown that PPARγ agonists attenuate apoptosis induced by cytokine or serum withdrawal. Survival promotion was attributed to PPARγ actions in cellular metabolic activities and the maintenance of T-cell mitochondrial membrane potential [485,486]. PPARγ further mediates T-cell differentiation. IL-17-secreting T helper cells (Th17) play a crucial role in autoimmune diseases. Their differentiation is induced by TGF beta/IL-6. PPARγ acts as a negative regulator of Th17 differentiation through inhibition of TGF beta/IL-6 signaling, and was not found to influence the differentiation of Th1, Th2, or regulatory T cells [487]. A recent elegant study employing a mouse model of atopic dermatitis evidenced that obesity exacerbated inflammatory responses through the conversion of a Th2-driven inflammatory disease to a worsened Th17-driven disease status. PPARγ expression was decreased in Th2 cells from obese animals compared to their lean counterparts. Using conditional deletion of PPARγ in T cells, the authors demonstrated the necessity of PPARγ to prevent uncontrolled Th17-mediated inflammation by redirecting the T helper cells towards a Th2 inflammatory response. Consequently, PPARγ agonists could reduce Th17-aggravated inflammation [283]. Interestingly, in colon cancer patients, the hierarchical clustering of a correlation matrix revealed that patients with high expression of the Th17 cluster had a poor prognosis. In contrast, no prediction of prognosis was associated with Th2 or Treg clusters, and enhanced Th1 clusters corresponded to better outcomes [488]. PPARγ agonists also inhibited allogeneic human memory T-cell responses in a model of human artery grafts in immunodeficient mice [489]. PPARγ is further involved in Treg homeostasis, as PPARγ deficiency led to reduced Treg recruitment in a colitis model [490], whereas PPARγ activation increased the induction of Tregs [491]. In general, PPARγ-expressing Tregs are considered to suppress adipose-tissue inflammation in obesity [492,493]. PPARγ activation in group 2 innate lymphoid cells (ILC2s) sustains type 2 cytokine production. Crucial to the pathogenesis of many allergic and fibrotic diseases, these cytokines can also promote tumorigenesis and cancer growth. Consequently, PPARγ deletion, specifically in ILC2s, reduced tumor growth in a mouse colorectal cancer model [494]. PPARγ expression in dendritic cells (DCs) was reported over twenty years ago. PPARγ activators were shown to inhibit the production of dendritic-cell IL-12, a strong Th1 pro-inflammatory inductor, thereby modulating the polarization of immune responses [495]. PPARγ activation provoked CD1d glycoprotein expression in DCs, leading to the selective induction of invariant natural-killer T-cell (iNKT cell) proliferation [496]. iNKT cells represent a distinct population of T lymphocytes, which have features of both conventional T cells and natural-killer (NK) cells and are considered important mediators of immune responses and tumor surveillance. PPARγ further enhances the anti-tumor efficacy of iNKT cells by assuring cholesterol synthesis and IFN-γ production in tumor-infiltrating iNKT cells [497]. A claudin-low subtype of bladder cancers has recently been described. They show an imbalance in decreased PPARγ expression and the resulting enhanced NF-κB signaling, and high cytokine and chemokine expression. These tumors are characterized by an enrichment of immune gene signatures but a simultaneous expression of immune-checkpoint molecules, which demonstrates that despite their high immune infiltration, they are also actively immunosuppressed [498]. However, increased PPARγ expression in bladder cancer through its suppression of NF-κB leads to the phenotype of immune cold tumors, which do not respond to immunotherapies and are characterized by low immune-cell trafficking, impaired T-cell activation, an abundance of myeloid-derived suppressor cells, and Tregs that release immunosuppressive cytokines [499]. Accordingly, in a different subtype of bladder cancer, muscle-invasive bladder cancer, recurrent mutations in RXRα lead to an imbalance of the PPARγ/RXRα heterodimer, and focal amplification of PPARγ. PPARγ overexpression impairs CD8^+^ T cell infiltration, possibly through NF-κB inhibition, and confers resistance to immunotherapies [500]. The important roles of PPARγ in affecting the immunophenotype of DCs, as well as how PPAR*γ*-regulated processes could be employed in the design of tumor vaccination strategies, are further reviewed in [501]. Immune tolerance of local DCs is believed to induce immune evasion and to contribute to the resistance of cancers to immunotherapies. In contrast to the anti-tumorigenic function of PPARγ in DCs proposed by many studies, Zhao and colleagues identified a paracrine Wnt5a-β-catenin-PPAR-γ signaling pathway driving FAO in DCs by which melanomas escape from immunotherapies. FAO promotes Treg-cell development and suppresses T-effector-cell activation. The blockade of FAO enhanced the effectiveness of anti PD 1 immunotherapy and slowed melanoma tumor progression [502]. DCs isolated from patients with advanced breast cancer expressed high levels of the adiponectin receptors AdipoR1 and AdipoR2. Using a different pathway to AdipoR1, AdipoR2 modified the inflammatory processes by activating the PPARγ pathway through the induction of COX 2. This leads to a blockade of NF-κB activation in DCs, and thereby attenuates their ability to stimulate antigen-specific T-cell responses [503]. High levels of Glutathione peroxidase 4 (GPX4), which inhibits ferroptosis, a lipid peroxidation-mediated cell death in tumor cells, are associated with poor prognosis in cancer patients. The inhibition of GPX4 with the compound RSL3 was shown to enhance the anticancer effect of cisplatin [504]. However, therapy-enhanced ferroptosis in dendritic cells severely impaired their anti-tumor functions that should produce cytokines, promote MCH expression, and activate T cells. It has been shown that PPARγ is responsible for RSL3-induced ferroptosis, which leads to the obstruction of DC maturation, as PPARγ knockdown was sufficient to restore anti-tumor activity in RSL3 treated dendritic cells [505]. Furthermore, PPARγ agonists impair innate immunity NK cell functions through inhibition of cytolytic NK activity [506]. The early identification of high PPARγ expression in the spleen [507] led many research groups to investigate its function in monocytes/macrophages. PPARγ has a fundamental role in lipid metabolism and is consequently highly expressed in foam cells, which are cholesterol-carrying macrophages in atherosclerotic lesions [508,509]. Following exposure to oxidized low-density lipoprotein, PPARγ is induced in monocytes and leads to the transcriptional induction of the immunotolerant state marker CD36, participating in atherosclerotic arterial lesion formation through its interaction with oxidized low-density lipoprotein (oxLDL), which triggers signaling cascades for inflammatory responses [508]. A series of studies investigated the anti-inflammatory effects of PPARγ thiazolidindione ligands, which were found to inhibit the inflammatory cytokines TNFα, IL-6, IL-1β [510], iNOS, MMP9, and scavenger receptor A (SR-A) [511]. PPARγ activation, therefore, mostly suppresses the immunoreactive state of a macrophage. However, non-thiazolidindione agonists of PPARγ failed to induce anti-inflammatory responses [512], and PPARγ-deficient embryonic stem cells could be differentiated into the monocytic lineage, suggesting PPARγ-independent effects of thiazolidindiones and 15d-PGJ2 on inflammation [513,514]. Nevertheless, PPARγ is important for defining the lineage of tissue-resident macrophages through transcriptional modulation in regulating the differentiation of pre-macrophages and alveolar macrophages, Kupffer cells, adipose-associated macrophages, and intestinal macrophages (reviewed in [27]); moreover, its activation primes primary monocytes for M2 differentiation, resulting in more pronounced anti-inflammatory activity in M1 macrophages [515]. In the setting of cancer, PPARγ activation was shown to reverse the MDSC and M2 macrophage-mediated suppression of the cytotoxic T lymphocyte (CTL) anti-tumor responses [516]. The deletion of PPARγ in macrophages further exacerbated mammary-tumor development in a mouse model. Mechanistically, PPARγ was found to suppress Gpr132 protein in macrophages, which is pro-inflammatory and tumorigenic [517]. The expression of PPARγ in macrophages favors an anti-inflammatory TAM phenotype. Macrophages exposed to breast cancer cell media achieved a TAM-like phenotype with features from both M1 and M2 polarization. The further addition of rosiglitazone to the breast cancer-conditioned medium reduced the secretion of M1 pro-inflammatory and pro-tumor M2-cytokines [518]. Similarly, the conditioned medium from macrophages exposed to apoptotic lung cancer cells inhibited the EMT, migration, and invasion of cancer cells. Apoptotic 344SQ activated PPARγ in macrophages, inducing enhanced phosphatase and tensin homolog on chromosome ten (PTEN) expression, which antagonized pro-tumorigenic phosphoinositide 3-kinase (PI3K) signaling [519]. However, PPARγ agonists were shown to drive the macrophage phenotype versus the M2 form in a model of a pathogen-induced macrophage challenge. This shift was accompanied by the enhanced production of TGFβ and arginase 1 and enhanced phagocytic activity [520]. Consequently, PPARγ activation in macrophages has been shown to fuel lung cancer progression and metastasis, especially through increased arginase 1 [342] and TGFβ1 [343] expression. Similarly, in a breast cancer model, PPARγ was found to induce M2 polarization through the induction of integrin β3 [346]. The cleavage of PPARγ by caspase-1 has been shown to enhance tumor promotion through the induction of TAMs. Truncated PPARγ translocates to mitochondria and interacts with medium-chain acyl-CoA dehydrogenase (MCAD), thereby inhibiting MCAD and FAO, which leads to lipid-droplet accretion and TAM differentiation. Caspase-1 deficiency significantly impaired tumor growth, underlining the importance of this pathway for tumor promotion by TAMs [345]. Highly interestingly, Moreira and colleagues demonstrated that CLAs, which are frequently used in dietary supplementation and known to activate PPARγ, have efficient anti-inflammatory effects that prevent colitis, but worsen colorectal cancer formation. CLAs induce macrophage- and T-cell-producing TGF-β via PPARγ activation, which enhances colorectal cancer progression. The macrophage-specific deletion of PPARγ abrogated pro-tumorigenic CLA effects in colon cancer [521]. In contrast to its overall anti-tumoral role in cancer cells, PPARγ governs major immuno-metabolic switches and alternative activation in immune cells, especially macrophages, thereby facilitating tumor initiation, progression, and metastasis. The PPAR functions and molecular mechanisms in cancer immunity are summarized in Figure 8.

## 10. Conclusions

Given the multiple diverse functions of PPARs in the cancer hallmarks, it is currently difficult to judge whether specific agonists or antagonists might have beneficial effects for cancer treatment. The effects in different cancer types and in each cancer type on stromal and tumor cells are divergent. Thus, with the advancement of personalized medicine, these differences should be considered for treatment decisions. In addition, research on dual- and pan-PPAR modulators might open new therapeutic strategies. The use and analysis of existing large databases, e.g., the National Veterans Health Administration (VHA) database including cancer patients with the coincidental administration of PPAR agonists, might give additional insights into the clinical role of PPAR modulation in cancer.

## Figures and Tables

**Table 1:** Effects of PPARα on cell proliferation and tumor growth.

Model	Intervention	Outcome	References
**In vitro**			
MCF-7, MDA-MB-231 breast cancer cell lines	Clofibrate, Wy-14,643	Proliferation⇧	[39]
MCF-7 breast cancer cell line	Leptin, glucose	Proliferation⇧	[40]
MDA-MB-231, MCF-7, BT-474 breast cancer cell lines	AA	Proliferation⇧	[41]
MDA-MB-231, MCF-7 breast cancer cell line	AA	Proliferation⇩	[42]
Triple-negative breast cancer cell lines	Fenofibrate	Proliferation⇩	[43]
SUM149PT and SUM1315MO2 inflammatory breast cancer cell lines	Clofibrate	Proliferation⇩	[44]
Ishikawa endometrial cancer cells	Fenofibrate	Proliferation⇩, tumor growth≈	[55]
BsB8 mouse medulloblastoma cells, human D384, and Daoy medulloblastoma cells	Fenofibrate	Proliferation⇩	[57]
U87 glioblastoma cell line	Fenofibrate	Proliferation⇩	[58]
Neuroblastoma cell line	Fenofibrate	Proliferation⇩	[59]
MDA-MB-231 breast, Panc-1 pancreatic cancer cell line	GW6471 (antagonist), Wy-14,643	Proliferation⇩ upon antagonist, proliferation⇧ upon agonist	[61]
A549 and SK-MES-1 lung cancer cell lines	Fenofibrate	Proliferation⇩	[64]
**In vivo**			
Mouse xenograft models	Fenofibrate	Tumor growth⇩	[43]
Wildtype mice	Wy-14,643	Liver tumorigenesis⇧	[47]
Hepatitis C virus transgenic mice with activated PPARα		Liver tumorigenesis⇧	[48]
Transgenic mice with PPARα activation in hepatocytes	Hepatocytic overexpression	Proliferation⇧	[49]
PPARα-knockout mice	Diethylnitrosamine-induced hepatocarcinoma	Liver tumorigenesis⇧	[52]
PPARα-knockout mice	Syngenic MEF/RS tumors, LLC1 lung cancer, B16 melanoma	Tumor growth⇩	[53]
PPARα-knockout mice	B16 melanoma	Tumor growth⇩	[69]
Ovcar-3 and Diss ovarian cancer cell lines, implanted tumors in nude mice	Clofibrate	Proliferation⇩, tumor growth⇩	[56]
PPARα knockdown in glioma stem cells, xenograft models	PPARα knockdown	Proliferation⇩, tumor growth⇩	[60]
Wildtype mice with LLC1 lung, B16 melanoma, or SKOV-3 ovarian cancer	NXT969 antagonist	Tumor growth⇩	[62]
KRasLA2 mouse model of spontaneous primary NSCLC, orthotopic lung cancer cell injection	Wy-14,643, bezafibrate	Tumor growth⇩	[65]
Wildtype and PPARα-knockout mice injected with Bcr/Abl-transformed B cells	Fenofibrate	Tumor growth⇩	[67]
HCT-116 colon cancer cell line, Xenograft model	Fenofibrate	Proliferation⇩, tumor growth⇩	[68]

⇧ Indicates increase, ⇩ indicates decrease.

**Table 2:** Effects of PPARγ on cell proliferation and tumor growth.

Model	Intervention	Outcome	References
**In vitro**			
Colon cancer cell lines	BRL 49653 activator	Proliferation⇩	[124]
Colon cancer cell lines	Troglitazone	Proliferation⇩	[126]
Liposarcoma cell lines	Pioglitazone	Proliferation⇩	[131]
Gastric cancer cell lines	Troglitazone, pioglitazone	Proliferation⇩	[132]
Gastric cancer cell lines	Troglitazone, 15d-PGJ2	Proliferation⇩	[133]
LA-N-5 nb neuroblastoma cell line	15d-PGJ2, GW1929	Proliferation⇩	[135]
SK-N-AS, SH-SY5Y neuroblastoma cell lines	Rosiglitazone	Proliferation⇩	[136]
U87MG, T98G glioblastoma cell lines	15d-PGJ2	Proliferation⇩	[137]
U87, U251 glioblastoma cell lines	Rosiglitazone	Proliferation⇩	[138]
A375 melanoma cell line	15d-PGJ2, ciglitazone	Proliferation⇩	[142]
Different melanoma cell lines	Multiple thiazolidinediones	Proliferation⇩	[140]
A375 melanoma cell line, xenograft model	Ciglitazone	Proliferation⇩	[141]
H1792 and H1838 NSCLC lines	Rosiglitazone	Proliferation⇩	[144]
H295R adrenocortical cancer cell line	Rosiglitazone, pioglitazone	Proliferation⇩	[145,146]
MCF-7 breast cancer cell line	15d-PGJ2	Proliferation⇩	[159]
MCF-7 breast cancer cell line	15d-PGJ2, ciglitazone	Proliferation⇩	[160]
MDA-MB-231, MDA-MB-453 breast cancer cell lines	C-DIM	Proliferation⇩	[164]
MDA-MB-231 breast cancer cells	Overexpression of PPARγ	Tumor growth⇩	[166]
MDA-MB-231 breast cancer cells	+ PPARγ-overexpressing fibroblasts	Tumor growth⇧	[166]
Leiomyoma cell line	Ciglitazone, troglitazone	Proliferation⇩	[161]
Ishikawa, Sawano, RL95-2 endometrial carcinoma cell lines	15d-PGJ2	Proliferation⇩	[148]
SKOV3 ovarian cancer cell line	C-DIM	Proliferation⇩	[149]
A2780, OVCAR3, OVCAR5, OVCAR8, OVCAR432, SKOV3, IGROV1 ovarian cancer cell lines	Ciglitazone, PPAR-γ antagonist GW9662	Proliferation⇩(agonist), proliferation⇧ (antagonist)	[150]
RPMI 8226 multiple-myeloma cell line	Overexpression of PPAR-γ	Proliferation⇩	[151]
B-cell lymphoma cell line	Silencing, overexpression of PPAR-γ	Proliferation⇧ (silencing), proliferation⇩ (overexpression)	[152]
G292, MG63, SAOS and U2OS osteosarcoma cell lines	Troglitazone	Proliferation⇧	[171]
143B, MNNG/HOS, MG-63, and TE-85 osteosarcoma cell lines	Troglitazone, ciglitazone	Proliferation⇩	[172]
H292, H3118, HMC1, HMC3A, HMC3B mucoepidermoid carcinoma cell lines	SR10221, SR2595, T0070907 inverse agonists	Proliferation⇩, tumor growth⇩	[174]
** In vivo **			
C57BL/6J-APC*^Min^*/+ mice	BRL-49,653, troglitazone	Tumor growth⇧	[122]
C57BL/6J-APC*^Min^*/+ mice	Troglitazone	Tumor growth⇧	[123]
Colon cancer cell lines, xenograft mouse model	Troglitazone	Proliferation⇩, tumor growth⇩	[125]
SW480 colon cancer cell line, xenograft model	C-DIM	Proliferation⇩, tumor growth⇩	[127]
A549 NSCLC line, xenograft models	Troglitazone, pioglitazone	Proliferation⇩, tumor growth⇩	[143]
NCI-H2347, NCI-H1993 lung adenocarcinoma cell lines, xenograft models	Pioglitazone	Proliferation⇩, tumor growth⇩	[173]
Huh7 and Hep3B hepatocellular cancer cell lines, xenograft models	Troglitazone	Proliferation⇩, tumor growth⇩	[147]
Dominant-negative mutant thyroid hormone receptor beta (TRbetaPV/PV mice)	Rosiglitazone	Tumor growth⇩	[157]
MMTV-VpPPARγ animals	Breeding with MMTV-PyV strain	Tumor growth⇧	[165]
MSE cell-specific PPARγ knockout (PPARγ-MSE KO)	7,12-dimethylbenz[a]anthracene (DMBA)-induced breast tumorigenesis	Tumor growth⇧	[167]
Thirty-eight patients with early-stage breast cancer	Rosiglitazone	Proliferation≈, tumor growth≈	[168]
UV and chemically induced skin carcinogenesis	Rosiglitazone, troglitazone	Tumor growth≈	[169]
CML LSCs Three patients with CML	Pioglitazone in combination with imatinib	Proliferation⇩, CMR≈5 years	[155]
EHMES-10, MSTO-211H mesothelioma cell lines, xenograft models	Troglitazone	Proliferation⇩, tumor growth⇩	[153]
Esophageal squamous-cell carcinoma line, xenograft model	Efatutazone; troglitazone	Proliferation⇩, tumor growth⇩; proliferation≈, tumor growth≈	[154]
Overexpression of dn PPAR-γ in myeloid lineage cells		Tumor growth⇧	[158]

⇧ Indicates increase, ⇩ indicates decrease.
